# Economic and Sustainability Impacts of Yield and Composition Variation in Bioenergy Crops: Switchgrass (*Panicum virgatum* L.)

**DOI:** 10.1021/acssuschemeng.3c05770

**Published:** 2024-01-22

**Authors:** Renee M. Happs, Rebecca J. Hanes, Andrew W. Bartling, John L. Field, Anne E. Harman-Ware, Robin J. Clark, Thomas H. Pendergast, Katrien M. Devos, Erin G. Webb, Ali Missaoui, Yaping Xu, Shiva Makaju, Vivek Shrestha, Mitra Mazarei, Charles Neal Stewart, Reginald J. Millwood, Brian H. Davison

**Affiliations:** † Renewable Resources and Enabling Sciences Center, National Renewable Energy Laboratory, Golden, Colorado 80401, United States; ‡ Strategic Energy Analysis Center, National Renewable Energy Laboratory, Golden, Colorado 80401, United States; § Catalytic Carbon and Transformation Center, National Renewable Energy Laboratory, Golden, Colorado 80401, United States; ∥ Environmental Sciences Division, Oak Ridge National Laboratory, Oak Ridge, Tennessee 37830, United States; ⊥ Institute of Plant Breeding, Genetics and Genomics, University of Georgia, Athens, Georgia 30602, United States; # Department of Crop and Soil Sciences, University of Georgia, Athens, Georgia 30602, United States; 7 Department of Plant Biology, University of Georgia, Athens, Georgia 30602, United States; 8 Department of Plant Sciences, University of Tennessee Knoxville, Knoxville, Tennessee 37919, United States; 9 Biosciences Division, Oak Ridge National Laboratory, Oak Ridge, Tennessee 37830, United States; 10 Center for Bioenergy Innovation, Oak Ridge National Laboratory, Oak Ridge, Tennessee 37830, United States

**Keywords:** feedstock variability, techno-economic analysis, life cycle analysis, switchgrass, bioethanol, minimum fuel selling price, composition, biomass yield

## Abstract

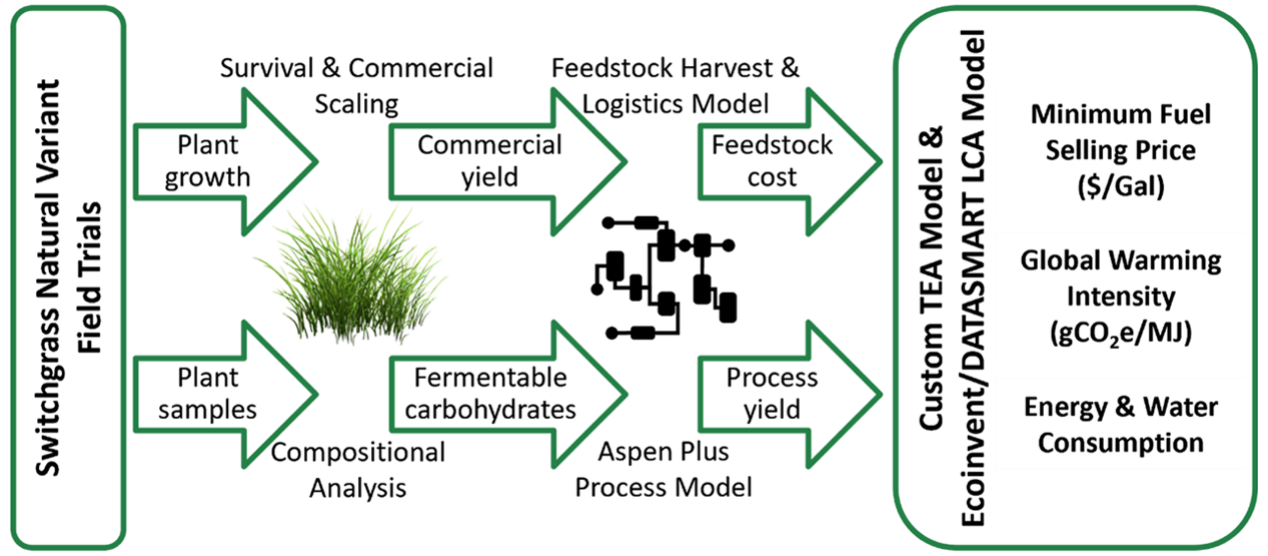

Economically viable production of biobased products and fuels requires high-yielding, high-quality, sustainable process-advantaged crops, developed using bioengineering or advanced breeding approaches. Understanding which crop phenotypic traits have the largest impact on biofuel economics and sustainability outcomes is important for the targeted feedstock crop development. Here, we evaluated biomass yield and cell-wall composition traits across a large natural variant population of switchgrass (*Panicum virgatum L*.) grown across three common garden sites. Samples from 331 switchgrass genotypes were collected and analyzed for carbohydrate and lignin components. Considering plant survival and biomass after multiple years of growth, we found that 84 of the genotypes analyzed may be suited for commercial production in the southeastern U.S. These genotypes show a range of growth and compositional traits across the population that are apparently independent of each other. We used these data to conduct techno-economic analyses and life cycle assessments evaluating the performance of each switchgrass genotype under a standard cellulosic ethanol process model with pretreatment, added enzymes, and fermentation. We find that switchgrass yield per area is the largest economic driver of the minimum fuel selling price (MSFP), ethanol yield per hectare, global warming potential (GWP), and cumulative energy demand (CED). At any yield, the carbohydrate content is significant but of secondary importance. Water use follows similar trends but has more variability due to an increased dependence on the biorefinery model. Analyses presented here highlight the primary importance of plant yield and the secondary importance of carbohydrate content when selecting a feedstock that is both economical and sustainable.

## Abstract 2

Switchgrass natural variant field results were used to evaluate how yield and composition differences affect economics and sustainability for biofuels.

## Introduction

A sustainable biobased economy requires the development and use of robust feedstocks with high yields and other desirable traits including tailored feedstock composition and abiotic/biotic stress resistance. Switchgrass (*Panicum virgatum*), a model herbaceous feedstock, has been the focus of research and development as a sustainable feedstock.^1^ Switchgrass has a broad native range in the U.S. and can be found across many temperate biomes, with higher productivity and ecosystem carbon storage than most conventional crops.^2^ Switchgrass is also desirable as a lignocellulosic crop as it can be grown on marginal lands otherwise unsuitable for food crops.^3^ However, switchgrass genotypes are typically adapted to only a narrow climatic range, and therefore, breeding has focused on selecting genotypes that produce high yield based on climate adaptation.^3^ Recent work on understanding the genetic basis of adaptation to different climates will assist with the development of high yielding cultivars targeted for different climatic zones.^4^

Producing dedicated energy crops at low costs depends on achieving high per-area yield rates to spread the many fixed costs of production (e.g., field preparation, nutrient application, etc.) across the greatest possible amount of biomass product. While there are large amounts of data on energy crop yields from small-scale breeding plots, experience with commercial-scale production is still limited. Small-scale plots may systematically overestimate the yield achievable at commercial production scales due to plot edge effects, the relatively high quality of land where many trials are located, and differences in biomass recovery efficiency between harvesting individual plants by hand versus large-scale mechanized harvest.^5^ For switchgrass, some studies show large differences between small plots and hectare-scale plantings,^5^ and others show no effect.^6^ Calculated per-area yield rates are systematically lower in gridded breeding plots compared to sward plots, though different varieties will perform better or worse under the competition of denser planting.^7^ There is currently no standard method for correcting planting density and estimating commercial-scale yield potential from breeding trial data.

Producing biofuels and other bioproducts from biomass is also sensitive to the chemical composition of that biomass. Lignocellulosic biomass from a nominal crop type can vary widely in cellulose, hemicellulose, lignin, ash, and moisture content depending on the crop variety, as well as environmental and management factors.^8^ Efforts to optimize biomass for biochemical conversion have often focused on reducing lignin content or adjusting lignin chemistry to decrease recalcitrance or facilitate greater solubilization of the carbohydrate fraction of the plant cell wall.^9^ In biochemical conversion, lignin in the cell wall creates a barrier to microbial, enzymatic, and even chemical deconstruction of fermentable sugars.^10^ Field studies of switchgrass have shown that genetically modifying cell wall characteristics via downregulating the caffeic acid O-methyltransferase (COMT) pathway in switchgrass leads to greater sugar release and ethanol yield^11^ along with a decrease in total lignin and alteration of the S/G ratio.^12^ Recent studies looking at environmental and climate change impacts on biofuel feedstocks have shown that drought stressed plants appear more recalcitrant to biochemical conversion, leading to lower fuel yields.^13^ Additional pretreatment steps may be required to overcome drought- or stress-induced plant recalcitrance.^14^ Finally, optimization of biochemical conversion is more likely to be cost-effective through using multiple avenues at once such as combining the use of less recalcitrant transgenic plants with pretreatment strategies.^15−17^

The wide genetic variability in natural populations of undomesticated (or partially domesticated) candidate bioenergy crops, such as switchgrass, provides an opportunity to select advantageous biomass properties. Previous work has identified phenotypic variation in switchgrass plant architecture, adaptation, growth, and cell wall compositional traits as analyzed in different switchgrass ecotypes and natural variants.^18−21^ Climate–gene–biomass associations were observed by Lovell et al. with the latitude from where a genotype originated being predictive of its survival in that region and of biomass production.^4^ Breeding efforts to improve feedstock biomass quality must simultaneously maintain the agronomic performance and yield of the feedstock crop.^9^

To understand the economic impacts of trait variations in switchgrass on a biorefinery process, techno-economic analysis (TEA) and life cycle assessment (LCA) need to be performed. TEA provides a means to understand both process-wide implications and economic impacts of parameter changes on a process. In the context of biorefineries, while several methods for these analyses exist, in this study, TEA is performed by utilizing an industrial-scale process simulation and then considering the cost of equipment, feedstock cost, additional operating expenses, and other economic considerations solve a discounted cashflow rate of return analysis for the minimum fuel selling price (MFSP). While growers will typically focus on biomass yield, biorefineries must focus on process yield, which can vary depending on both the type of biomass and the type of conversion. For this study, the purpose of a TEA is to bring both grower and biorefinery goals together under one metric, namely, fuel yield per land area per year. Additionally, the MFSP is regarded as an indirect measure of feedstock quality while considering all major economic drivers, including potentially heavy operating and capital costs. Previously, TEA models constructed using growth and compositional data showed the importance of feedstock composition as an important but secondary factor after yield in the MFSP in *Populus trichocarpa*.^22^ This work used compositional and yield data from several hundred natural poplar variants planted in common gardens and grown under the same conditions for up to seven years.

Building on our previous work on *Populus*,^22^ here we conduct TEA on a natural variant population of switchgrass and also apply a cradle-to-biorefinery-gate attributional LCA to estimate associated environmental impacts. LCA quantifies material and energy inputs to the biofuel and feedstock production processes, as well as the associated emissions and environmental impacts, and is a crucial tool to use in concert with TEA. Our LCA quantifies the variability in ethanol life cycle global warming potential (GWP), cumulative energy demand (CED), and Available Water Remaining (AWARE) indicator due to variability in on-farm yield and fermentable carbohydrate mass fraction (FCMF) from the switchgrass natural variant population.^23^ GWP and CED values are compared with extant literature values as a way of validating the results of this study. AWARE is important due to water use in the biorefinery, even though the switchgrass was rain-fed. Ultimately, this work provides a comprehensive analysis of switchgrass as a biofuel feedstock and will allow feedstock producers to choose a crop that has been bred to complement climate, land use, and biorefinery economics.

## Results

### Switchgrass Yield and Cost

**Figure 1:**
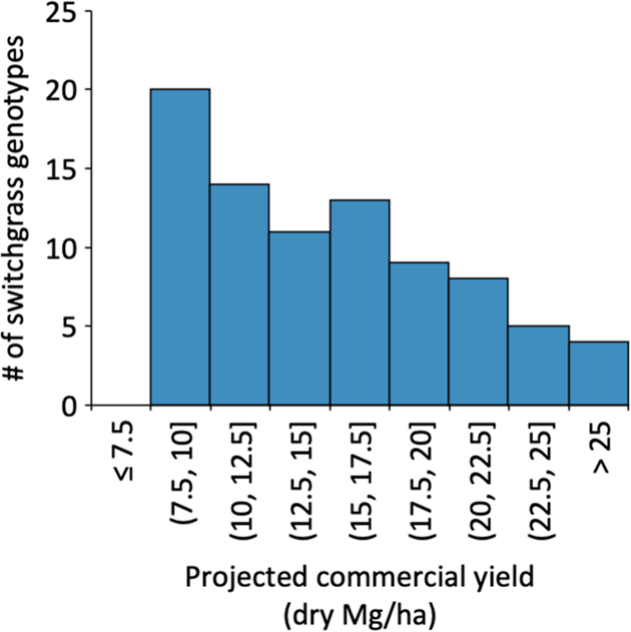
Histogram of estimated commercial-scale yield rates > 7.5 Mg/ha for switchgrass genotypes.

Our estimates of commercial-scale yield potential for the different genotypes in the switchgrass natural variant population are shown in Figure 1. About a quarter of the original natural variant genotypes (i.e., 84 of 331) showed survival of all replicates across the three common garden sites (Tifton, GA; Watkinsville, GA; Knoxville, TN), minimal between-replicate variability, and a projected commercial-scale yield potential greater than 7.5 dry Mg/ha. Less than half of the genotypes in this collection achieve a commercial-scale yield above 15 dry Mg/ha, and only one in five (17 genotypes) achieves > 20 dry Mg/ha. These three common garden sites are in hardiness zones 7b (Knoxville, TN), 8a (Athens, GA), and 9a (Tifton, GA). Most upland genotypes, which are adapted to the northern U.S., experienced mortality or low yields in our common gardens, so the 84 genotypes included in Figure 1 and subsequent analyses are weighted toward lowland and coastal ecotypes. For comparison, AP13 — a clonal genotype from the Alamo switchgrass landrace and the first switchgrass genotype sequenced^4^ — had an estimated commercial yield of 4.9 Mg/ha in these trials and thus was not included in further analyses.

Typical perennial switchgrass cultivation involves planting with yield increases over several yields. Postsenescent harvesting by mowing and baling generally maximizes the sustainability. Baled switchgrass is stored either on farm or after transport to the biorefinery, where it is further chipped before conversion.

**Figure 2:**
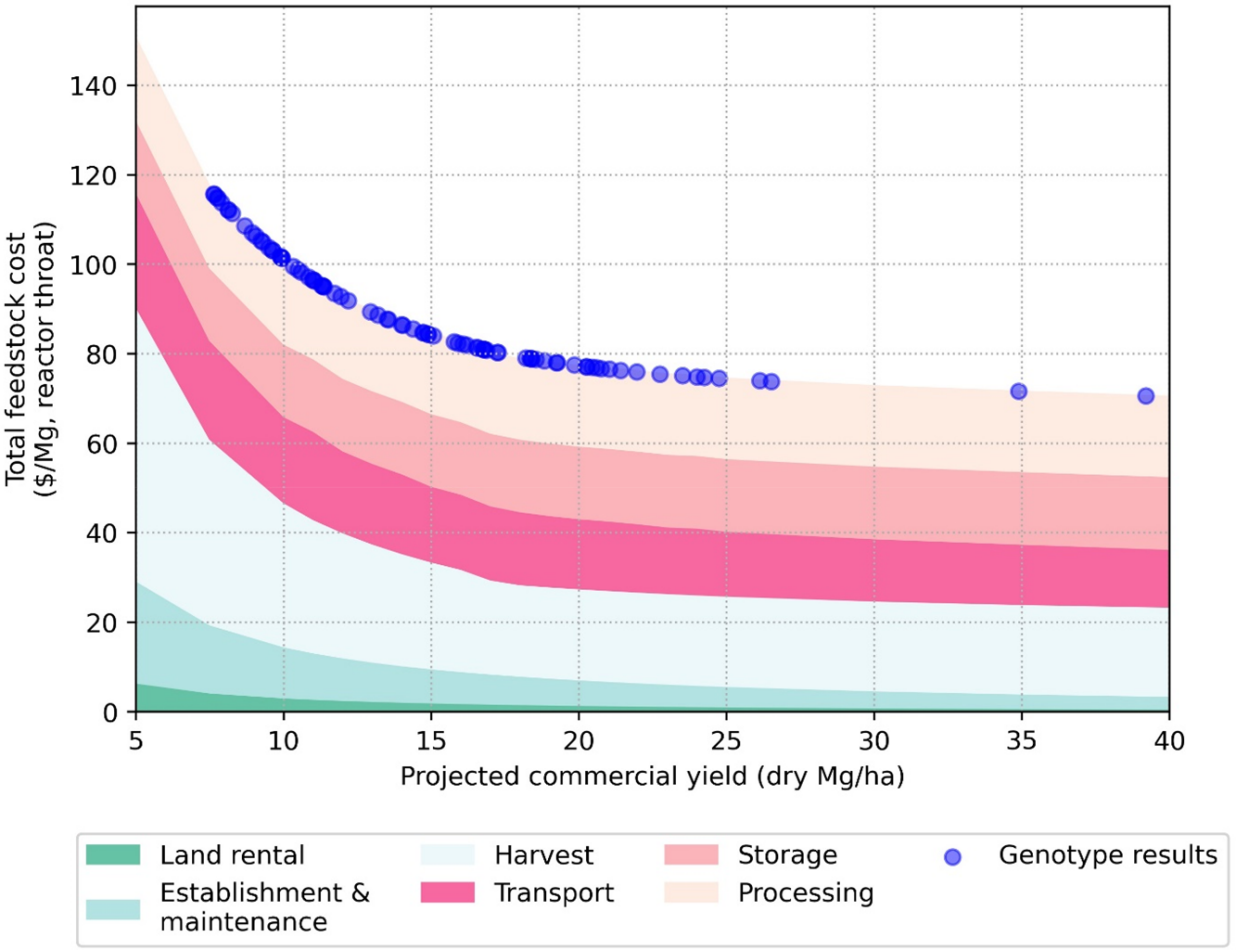
Delivered switchgrass costs including production (land rental, establishment, and maintenance), harvest, storage, transport, and preprocessing at the biorefinery. Shaded areas show the results of the cost model over the range of yields observed; purple dots show estimated costs for the 84 specific genotypes selected from the natural variant population for this study.

In order to estimate the delivered cost of switchgrass in an industrially relevant supply chain, a harvest and logistics cost model was executed for the range of yields in Figure 1. The model considers the costs of production (land, planting, and annual maintenance), harvest (mowing, baling with a large rectangular baler, and in-field transport to field edge), storage, transport, and preprocessing (milling or chipping) at the biorefinery. Figure 2 summarizes these results for each of the selected genotypes and indicates the contribution of these inputs. Figure S1 plots these model estimates separately based on the estimated biomass yield. Harvest, transport, and land maintenance costs decrease as the switchgrass yield increases from 5 to 17 dry Mg/ha. An increase in yield reduces the land requirement, which affects the harvest and land maintenance cost, while the reduced supply shed footprint affects the transport cost. However, total costs become much less sensitive to yield above 18 dry Mg/ha, with less than two percent change in total cost per megagram when increasing the yield by 1 Mg per hectare. It should be noted that this metric is not the price that a biorefinery would pay for feedstock at the refinery gate but rather a techno-economic cost that would be incurred if the refinery managed the feedstock supply chain up to the beginning of the conversion processes or at the “reactor throat.” We assumed that if the refinery was purchasing the feedstock from local growers, then the purchase cost would be based on the incurred cost plus a factor of grower profit.

For the purposes of this study, potential compositional variability that can be introduced during harvest, handling, and storage is neglected, and optimal management practices are assumed. While this does neglect key information that would be needed by a biorefinery in designing a feedstock supply chain, it is an acceptable simplification for assessing biological variability, the focus of this study. Costs range from $70/dry Mg^–1^ for the highest-yielding genotypes to almost $120/dry Mg^–1^ for the lowest yields at our cutoff of 7.5 dry Mg/ha; genotypes with lower yields would likely not be of commercial interest in the region. These costs are comparable to those estimated by Womac et al. (2018) for a yield of 17 Mg/ha based on field trials in East Tennessee.^24^ Delivered cost decreases sharply as yield increases, showing a clear relationship to aid selection of the most economically attractive genotypes.

### Cell Wall Composition in Natural Variant Panel

The 331 natural variant switchgrass genotypes from the Watkinsville common garden panel were analyzed for structural carbohydrate (sugar) and lignin using nuclear magnetic resonance (NMR) spectroscopy and pyrolysis molecular beam mass spectrometry. ^1^H NMR was used for high-throughput analysis to analyze hydrolysate samples generated in duplicate as described previously.^22^ Partial Least Squares models for four monomeric sugars (glucose, xylose, galactose, and arabinose; mannose is not present in switchgrass) were built using high performance liquid chromatography (HPLC) determined concentrations from a model sample set to predict sugar composition in NMR spectra of hydrolysates.

**Figure 3:**
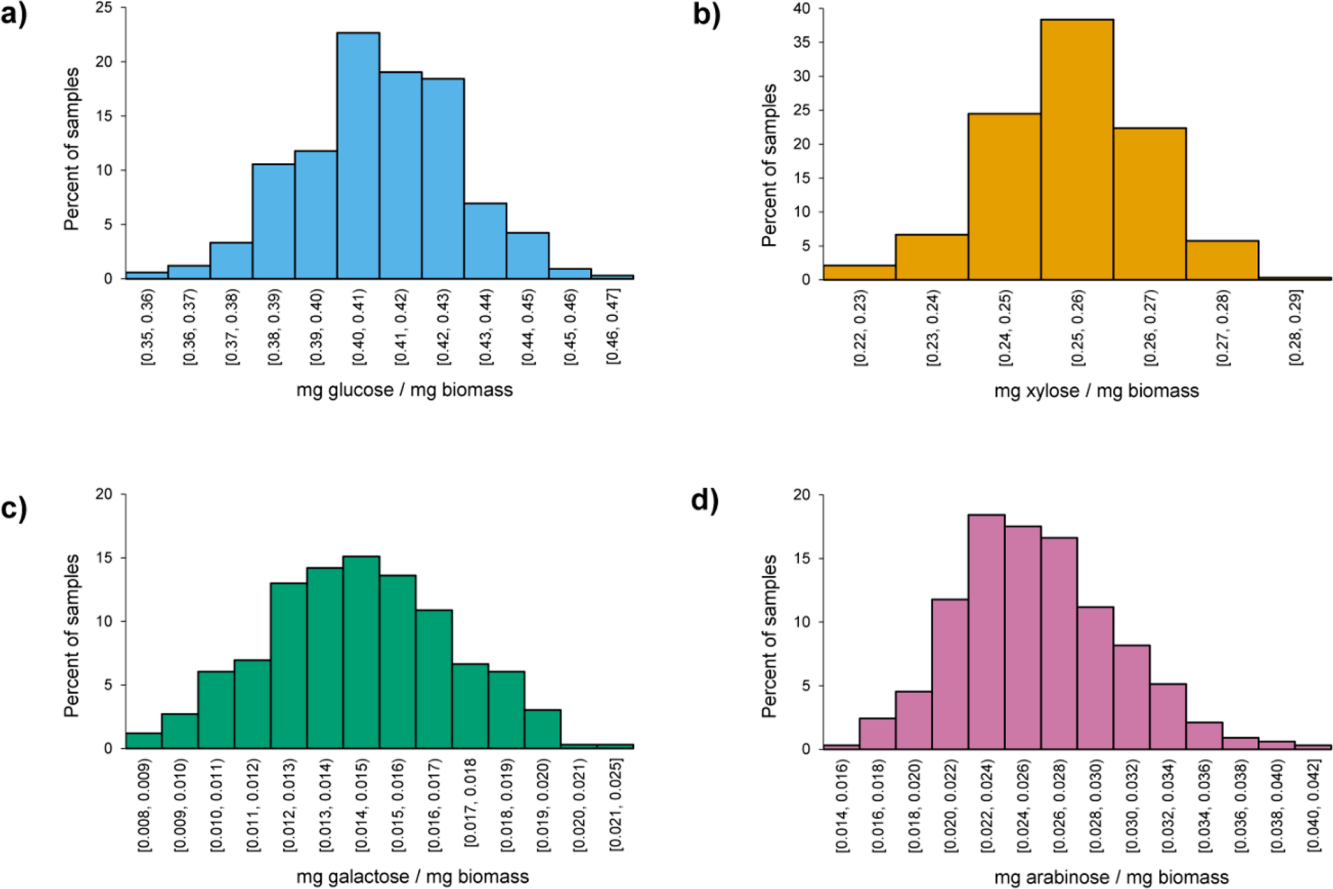
Frequency distribution (*n* = 331) of sugar composition across the switchgrass natural variant population. Bin width was calculated using Scott’s Normal Reference Rule for each monomeric sugar: (a) 0.01 mg glucose mg biomass^–1^; (b) 0.01 mg xylose mg biomass^–1^; (c) 0.001 mg galactose mg biomass^–1^; (d) 0.002 mg arabinose mg biomass^–1^.

Figure 3 gives the histogram plots of estimated glucose, xylose, galactose, and arabinose for the natural variant switchgrass sample set based on the anhydrous biomass solids. Bin widths were calculated using Scott’s Normal Reference Rule. Table S1 presents the overall averages and statistics of the sample set.

There is variation in the glucose and xylose ranges, with values of more than ±1 standard deviation from the average of the set and glucose having a broader distribution than xylose. Unlike our previous *P. trichocarpa* analysis, this switchgrass population has greater variation in the two detected minor sugars, galactose and arabinose. Previous analysis of switchgrass composition found higher values of arabinose than reported here, although those values were only among a handful of a small subset of switchgrass cultivars.^26,26^ Additionally, the glucose values reported here are higher than reported previously, but the xylose values in the natural variant population have a similar range as some previous studies of whole plants but a lower range than a study that used stems only.^25,26^ Generally, the switchgrass natural variant population has more samples close to the average values, with less plants on the extreme end of variation ranges than we saw previously in our poplar study.^22^ The current study did not consider leaf to stem ratios, and it has been shown previously that structural sugars vary between stems and leaves in grasses.^25^

**Figure 4:**
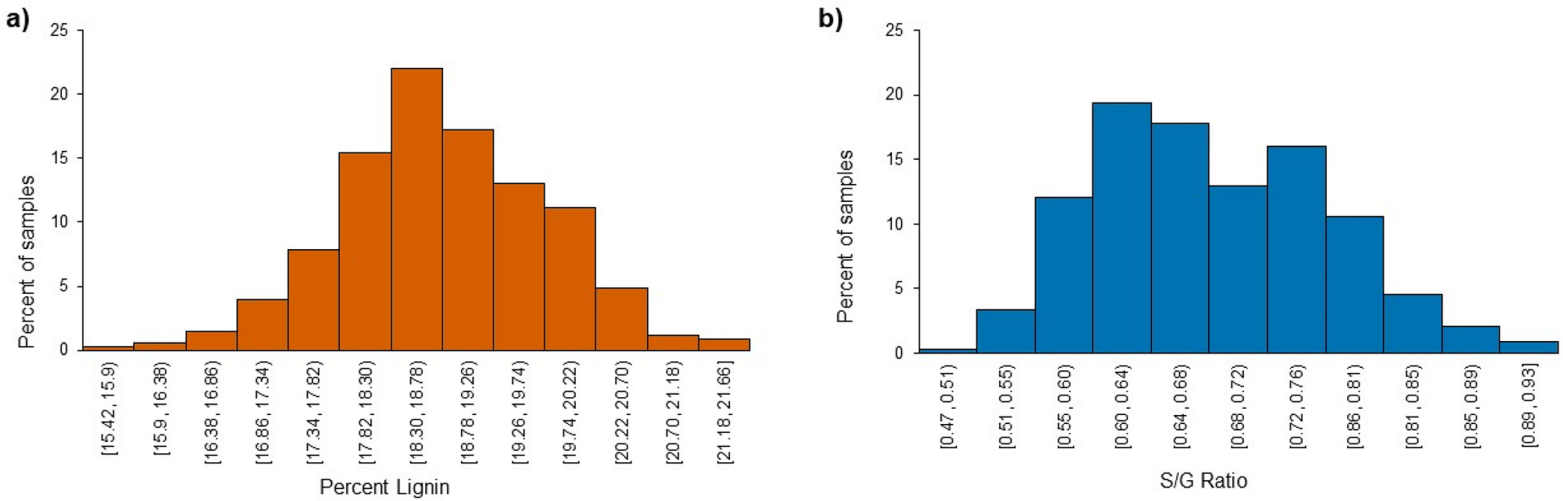
Frequency distribution (*n* = 331) of percent lignin and S/G ratios across the switchgrass natural variant population. Bin width was calculated using Scott’s Normal Reference Rule: a) 0.48 for % lignin; b) 0.08 for S/G ratio.

Figure 4 shows that lignin content in the natural variant population varied between 15 and 21% (w/w) (ranging from 0.15 to 0.21 mg/mg biomass dry weight) with an average of 19% (±1%) (w/w). The monomeric ratio of the lignin polymers (S/G ratio) varied from 0.47 to 0.91 with an average of 0.68 (±0.08). These lignin trait values are consistent with those reported for switchgrass elsewhere; overall variability of the lignin traits is lower in the switchgrass common garden in comparison to poplar natural variants and pedigrees.^22,27^ The S/G ratio in switchgrass may be related to biomass productivity and sustainability traits as well as conversion processing yields and product distributions but is not included in this analysis.^28,29^

### Techno-Economic Analysis

**Figure 5:**
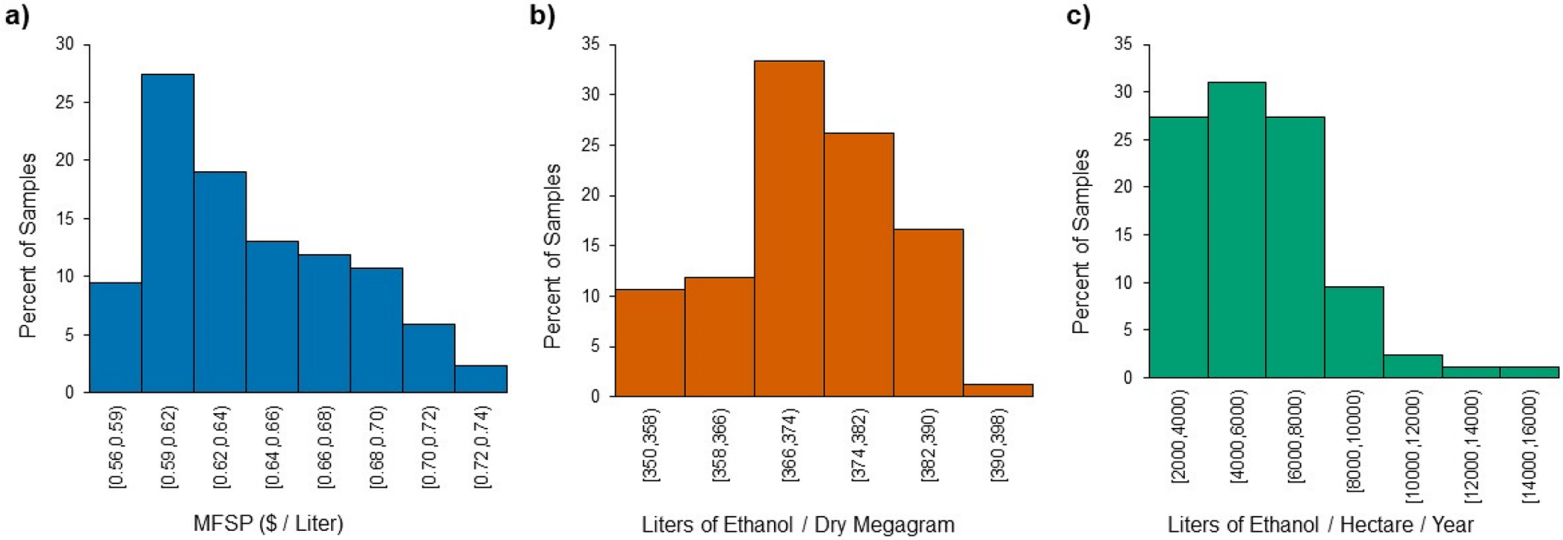
Frequency distribution for a switchgrass subset (*n* = 84) incorporated in the techno-economic analysis highlighting economic and yield metrics: a) MFSP, b) process ethanol yield, and c) field ethanol yield.

Frequency distributions for the 84 switchgrass samples used in process modeling are shown in Figure 5 for a) MFSP: x̅ = $0.64/L, σ = $0.04/L (6.04%), b) Process ethanol yield: x̅ = 372 L/dry Mg, σ = 10 L/dry Mg (2.63%), and c) field ethanol yield: x̅ = 5,700 L/ha/year, σ = 2,320 L/ha/year (40.6%).

**Figure 6:**
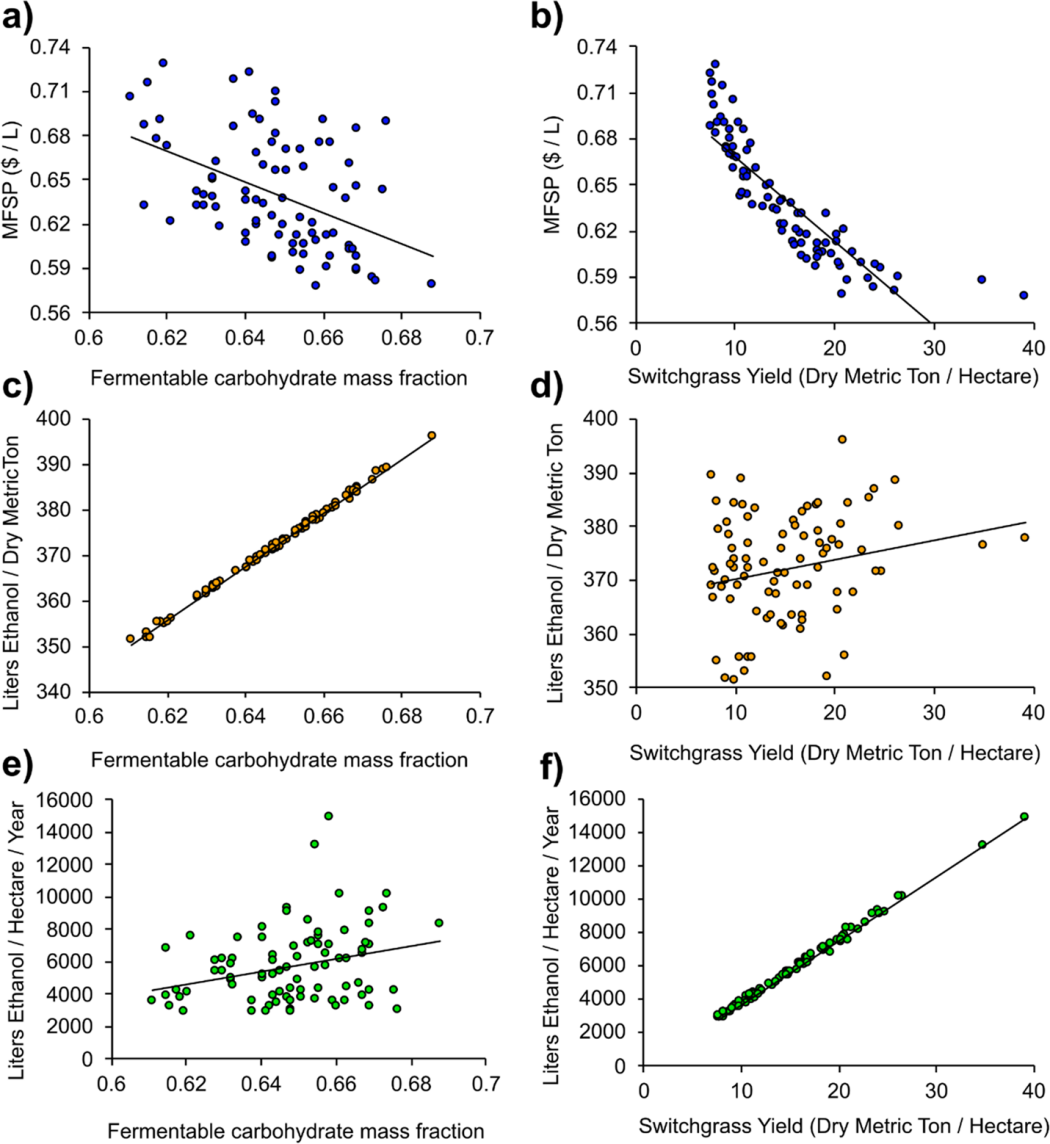
Relationships between (a, b) the MFSP ($/L), (c, d) process ethanol yield (L/dry Mg), and (e, f) field ethanol yield (L/ha/year) against the mass fraction of fermentable carbohydrates (glucan, xylan, arabinan) and switchgrass yield. Linear trends are observed for the MFSP and field ethanol yield against switchgrass yield and process ethanol yield against the fraction of fermentable carbohydrates in the switchgrass samples. *R*^2^ values: (a) 0.207, (b) 0.744, (c) 0.997, (d) 0.050, (e) 0.080, and (f) 0.996.

**Figure 7:**
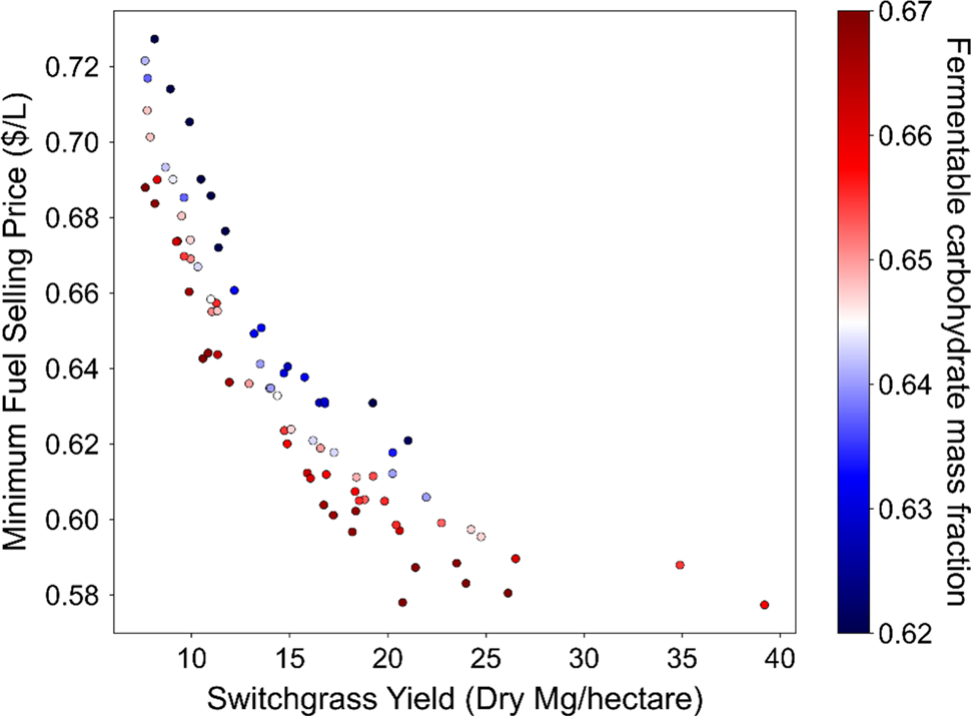
MFSP ($/L) plotted against switchgrass yield (dry Mg/ha) with the mass fraction of fermentable carbohydrates (glucan, xylan, and arabinan) shown for each sample by color. The MFSP is most strongly influenced by switchgrass yield, decreasing as yield increases, while the range of the MFSP for a given switchgrass yield trends toward being driven by the mass fraction of fermentable carbohydrates with higher fractions of glucan, xylan, and arabinan (red) toward the lower end of the MFSP and lower fractions (blue) toward the higher end of MFSPs.

To determine potential drivers behind the MFSP, process ethanol yield, and field ethanol yield, each was plotted against either fermentable carbohydrate mass fraction or switchgrass yield (Figure 6). These data are combined into Figure 7 which plots the MFSP versus estimated switchgrass yield with the carbohydrate composition indicated by color (data from Figure 6a). We note that in the process model used, conversion to ethanol is only directly influenced by the fermentable carbohydrates; lignin quality or other compositional phenotypes are not included in this basic biorefinery model. Figure 6d shows the plot of the two important yields–switchgrass yield (Mg/ha) versus conversion yield (L ethanol/Mg)–which are poorly correlated. Due to the high correlation seen in Figure 6c of conversion yield versus carbohydrate mass fraction, a plot of carbohydrate mass fraction versus switchgrass yield appears almost identical to that in Figure 6d. Due to the multiple assumptions, these values should only be used for genotype comparison not as an absolute economic value. Given the higher the yielding genotypes (estimated to be >7.5 dry Mg/Ha), the primary economic importance of switchgrass biomass yield is clear.

### Life Cycle Assessment

Numerical LCA results (GWP, CED, and AWARE) for each switchgrass variant are given in Table S2 alongside the fermentable carbohydrate mass fraction, on-field yield, and MFSP values. Statistical summaries of the GWP, CED, and AWARE values observed in this study are given in Table S3.

GWP values observed range from 466–586 g CO_2_e/L, with a mean of 517 g CO_2_e/L, and CED values range from 8.70–10.5 MJ/L, with a mean of 9.50 MJ/L. The GWPs observed align with values reported previously.^30^ Both the GWPs and CEDs observed are substantially above analogous values obtainable from the 2021 Greenhouse gases, Regulated Emissions, and Energy use in Technologies (GREET) model^31^ (CED was not reported in Nocentini et al.^30^). That GWP in this study is higher than that calculated with GREET is likely due to the exclusion in this study of any agriculture-related greenhouse gas sequestration, soil carbon changes, or other land use change impacts, since this is a general model that is not associated with a specific location or soil. GREET includes impacts from land use change that for switchgrass result in a net GWP reduction. The current study also uses variant-specific inventories of agricultural inputs and biorefinery inputs that generally differ from the default inventory for switchgrass ethanol provided with GREET, which likely contributes to the higher CED and GWP values observed. Tables S4 and S5 provide comparisons of these inventories. The AWARE indicators observed in this study range from 45.3–51.7 m^3^/L with a mean of 48.3 m^3^/L. To our knowledge, there is no extant work applying the AWARE indicator to a comparable switchgrass ethanol life cycle.

In addition to total life cycle impacts for each variant, a process contribution analysis (PCA) was also performed for selected variants that represent the full range of yield and fermentable carbohydrate mass fraction (FCMF) values in this study. Details of which variants are included in the PCA are given in Figure S2. The PCA was done to identify any trends that exist in process-specific impacts due to variability in either FCMF or in switchgrass yield. A description of the PCA methods is given in the Process Contribution Analysis section in the SI. Graphical PCA results are given in Figures S3, S4, and S5, and numerical PCA results are given in Tables S6 and S7. Overall, increased switchgrass yield corresponds to decreased impacts in each process category, while increased FCMF does not correspond uniformly to decreased or increased impacts in each process category.

**Figure 8:**
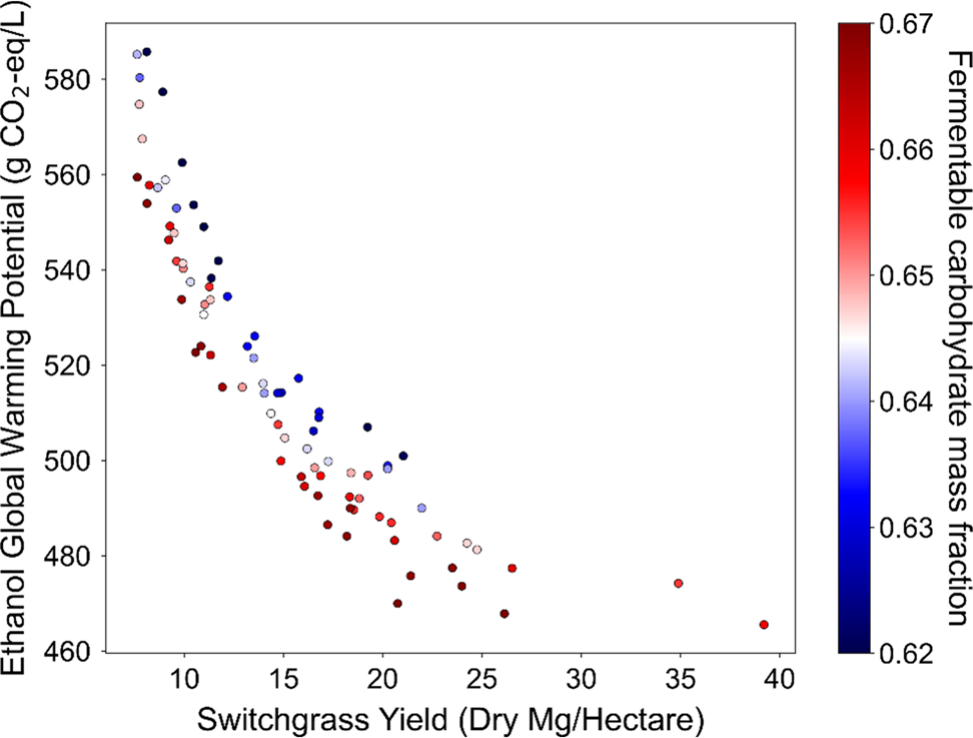
Global warming potential (g of CO_2_e/L of ethanol) shows a strong negative correlation (*R*^2^ = 0.76) with switchgrass yield (dry Mg/ha). Higher FCMF is generally associated with lower GWP among variants with similar yields.

GWP (g of CO_2_e/L) is plotted against annual switchgrass yield (dry Mg/ha) in Figure 8 with color indicating the FCMF of each variant. The overall trend is an increased switchgrass yield corresponding to decreased GWP (*R*^2^ = 0.76). This is because as yield per acre increases, the total amount of land that must be cultivated to supply the biorefinery decreases, resulting in overall lower impacts from the agricultural operations in the life cycle. Among variants with similar yields, increased FCMF corresponds to lower GWP. The variability in FCMF is roughly constant across the range of observed yields.

CED (MJ/L) is plotted against the switchgrass yield in Figure S6, with FCMF again indicated by color; this figure appears similar to Figure 8. Increased switchgrass yield corresponds to a lower CED, with an *R*^2^ of 0.77. Increased FCMF generally corresponds to lower CED among variants with similar yields, with this trend being most pronounced for moderate yields and less pronounced at high yields. Both trends are analogous to the trends seen for GWP and the MFSP, because both the LCA and TEA results are derived largely from the quantity of material and energy inputs used to produce switchgrass and ethanol.

**Figure 9:**
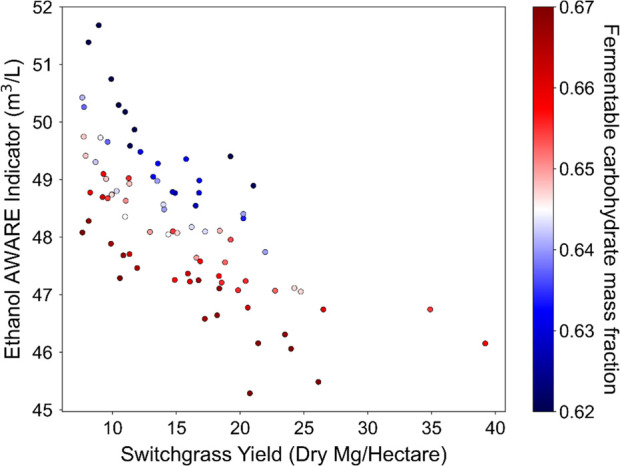
Ethanol Available Water Remaining indicator (AWARE, m^3^/L) shows some negative correlation with the switchgrass yield (*R*^2^ = 0.49). Increased FCMF corresponds to lower AWARE values among variants with similar yields.

Finally, the AWARE indicator (m^3^ water/L) is plotted against the switchgrass yield and FCMF in Figure 9. There is again a trend of decreasing AWARE with an increasing yield, although the trend is significantly weaker than for GWP or CED (*R*^2^ = 0.49). A higher FCMF also corresponds to a lower AWARE value for variants with similar yields, as was the case for both GWP and CED. Much of the AWARE indicator value is due to the production of inputs to the biorefinery (as shown in the PCA results, Figure S5) as the switchgrass growth is assumed to be rain-fed. Biorefinery inputs vary primarily with FCMF, leading to increased variability in AWARE indicator values relative to CED and GWP values, which depend more heavily on switchgrass agriculture and logistics than on the biorefinery.

## Discussion

The field ethanol yield is very strongly correlated to the switchgrass yield (*R*^2^ = 0.996), demonstrating that the switchgrass yield is the most important factor in maximizing the amount of ethanol produced for any given land area; these data were drawn from the 84 higher yielding genotypes with an estimated associated per-area biomass yield of 7.5–39 Mg/ha. There is a strong trend of the process yield (Figure 6c, L ethanol/dry Mg switchgrass) versus the mass fraction of fermentable carbohydrates (*R*^2^ = 0.997), which may be expected as fuel yields are a function of sugar concentration. This process ethanol yield varies slightly due to the composition of the individual fermentable carbohydrates and minor changes in the ethanol yield for each specific fermentable sugar. The overall field ethanol yield, however, showed no correlation to mass fraction fermentable carbohydrates (*R*^2^ = 0.05) despite the field ethanol yield being derived from a combination of the process ethanol yield and switchgrass yield.

There is a weak trend when using all of the data for the MFSP against the fermentable carbohydrate mass fraction overall. This trend is much stronger if one limits the consideration to any particular yield value. For example, when considering only the higher yielding switchgrass variants (≥20 dry Mg/ha), there is a clear trend of the decreased MFSP with the increasing fermentable carbohydrates (see Figure S7). For these high yielding genotypes, an incremental increase in the biomass yield may have a similar economic impact as an incremental increase in fermentable carbohydrates. There is a wider degree of natural variation among genotypes in the switchgrass field yield (from <7.5 to 39 dry Mg/ha) than in the switchgrass compositional quality (from 0.62 to 0.67 mass fraction of fermentable carbohydrates). The data suggests that the biomass yield and fermentable carbohydrate composition are largely uncorrelated by genotype. This implies that there is the possibility to breed or engineer switchgrass to improve both traits at the same time.

For the LCA results, the conclusions are broadly similar. Here, we utilize GWP, CED, and AWARE as indicators of important sustainability outputs for CO_2_ release, energy use, and water use. The pattern observed for GWP is almost identical with that of the MFSP, with yield being the most important, followed by quality (as indicated by the blue-to-red color shift in fermentable carbohydrates at any specific yield value in Figure 8). There are diminishing returns observed at the highest yields: the reduction in impacts with increased yield decreases as yield increases beyond approximately 25 dry Mg/ha. The tight relationship between the TEA and LCA results is expected because both assessments are based on quantities of the various material and energy inputs into the feedstock and fuel production process. TEA and LCA results might start to diverge if the LCA were expanded to include soil carbon changes or nitrous oxide emissions, nonengineered processes that show a great deal of unavoidable biological variability.^32^

Both GWP and CED fall by about 20% over the range of switchgrass yields. This analysis points out the importance of considering a range of the most critical parameters (e.g., yield), as the insights gained from the GWP and CED values of individual variants are much more insightful than the mean values, as shown in Table S3. AWARE only falls by about 10% over the range of biomass yields. This is partly because water use is more closely associated with the biorefinery inputs than the feedstock production inputs. This points out a limitation of much prior work that tends to use a single parameter for the analysis as representative of an entire crop species or limits the sensitivity analysis to a single biorefinery parameter at a time.^33,34^

In this study, the 84 genotypes included in the full analysis were predominantly lowland and coastal genotypes, as most of the upland genotypes had lower survival when grown in the southeast U.S. Further studies may show other advantages for additional switchgrass variants or other feedstocks better suited for other regions. We note that the MSFP or LCA values presented should be used for initial comparison of genotypes and assumptions for further study. Plant breeders, growers, and biorefineries have many factors to consider when choosing a feedstock, and understanding the variation in both growth and compositional phenotypes, as well as the resulting variation in environmental impact, is essential. Based on the observed field ethanol yield, on average, switchgrass requires less land area than poplar to produce an equivalent amount of ethanol than poplar. The TEA trends for switchgrass (this paper) and for poplar^22^ show that yield is the most important factor but has less incremental impact at the highest yields. Like poplar, for any given switchgrass yield, the MFSP looks to be determined by composition quality. The compositional impact on cost and LCA is somewhat less for switchgrass than that for poplar. Still, within a yield range, composition starts to carry importance. Therefore, when growers need to make a choice between multiple high yielding accessions that are suitable for cultivation in their growing region, they should consider feedstock quality as a secondary criterion. It seems that for truly sustainable feedstock both yield and composition matter.

## Conclusions and Future Directions

This analysis shows the critical and primary importance of the overall biomass yield as a determinant of ultimate fuel cost and environmental impacts. However, for variants with similar yields, these impact factors tend to be ranked by composition (the amount of fermentable carbohydrates). It also shows the value of exploring natural variation in largely undomesticated feedstocks, such as switchgrass. These common gardens are being used for genome-wide association studies.^35,36^ Process-specific updates and additional feedstock quality aspects such as ash/silica which influences milling and chipping costs or lignin composition, which influences possible valorization, were not considered here (where lignin was just used for heat) and would increase the influence of composition. These compositional and process improvements (especially for lignin) should be the subject of future efforts. Likewise higher glucan genotypes would have slightly higher fuel yields, as the glucans are currently more easily converted. The potential for other factors to directly influence the results were not considered–these include known inhibitory effects of drought-stressed switchgrass.^14,37^ However, we expect biomass land yield to remain dominant except to distinguish among the highest biomass producing variants.

Switchgrass yield is the primary factor in both the TEA and LCA metrics. This needs to be used as a primary driver for the selection and field testing of natural variants. The range of these variants grown under common conditions and their consistent yield performance at different latitudes in the Southeast indicate strong genetic determinants. Razar et al. (2022) observed QTLs related to switchgrass yield^38^ that can be targeted in breeding. Here, we show the value of considering variation as an opportunity to improve the biorefinery system–not as a risk to be avoided. Further TEA and LCA studies should start to examine feedstock yield variability in the context of environmental effects such as drought, location-specific effects, switchgrass stand age, land management decisions such as the amount and timing of fertilizer application, and process variability. These studies may be used to inform decisions in precision agriculture and improve the accuracy of LCA impacts and indicators such as GWP.

## Materials and Methods

### Switchgrass Natural Variant Diversity Panel and Tissue Sampling

A core switchgrass diversity panel consisting of 331 genotypes, of which a majority were provided by Thomas Juenger at the University of Texas-Austin (as described in Lovell et al.^4^), was propagated at the University of Georgia in 2018 and 2019. The panel is comprised exclusively of tetraploid genotypes, with a bias toward southern adapted ecotypes (35% lowland, 41% coastal, 24% upland). Four replicate panels were established at Knoxville, TN (Spring 2019) (35.903094, −83.959253), six at Tifton, GA (four in Summer 2018 and two in Spring 2019) (31.438345, −83.580185), and two at Watkinsville, GA (one in Spring 2019 and one in Summer 2020) (33.721096, −83.310268). Field layout for the Knoxville and Watkinsville panels followed Lowry et al.,^20^ using a honeycomb design (3.5 and 3 m between linear plants, respectively), Dewitt weed cloth, and cultivar Blackwell border plants. The Tifton panel had a 0.9 m grid layout without weed cloth. In late fall/early winter of 2019 and subsequent years, entire plants were harvested at 10 cm above ground level and weighed; for nearly all cultivars, this was postsenescence. A subsample from each plant was chipped, weighed, and dried at 60 °C. Dried samples were weighed for dry mass correction. 319 genotypes from the 2019 Watkinsville panel were processed using a Wiley #4 mill and 1 mm screen for chemical analysis at NREL. More details on the use of these populations will be communicated in a future paper.

### Biomass Quality Analysis

#### Hydrolyzate Preparation

Prior to hydrolysate preparation, samples were destarched, and ethanol was extracted to remove starch, free sugars, and extractives not related to structural cellulose, hemicellulose, or lignin. The NREL laboratory analytical procedure “Determination of Structural Carbohydrates and Lignin from Biomass” was scaled down as described previously.^22,39,40^ Samples were stored for up to 1 week at 4 °C before being filtered prior to preparation for NMR analysis.

#### NMR Parameters

Liquid hydrolysates were prepared as reported previously.^22^ Briefly, a D_2_O stock solution was added to hydrolysates for a final concentration of 0.01 mg mL^–1^ TSP-*d*_4_ (Cambridge Isotope Laboratories, Andover MA, USA) used for chemical shift reference. ^1^H spectra were collected at 25 °C with a Bruker 5 mm BBO probe using NOESY 1D presaturation to suppress the water peak, 64 scans, and a 5 s recycle delay. A SampleJet automatic sample changer with 96-tube racks was used for high-throughput analysis on a Bruker Avance III spectrometer (Bruker Bio-Spin, Billerica, MA, USA) at 14.1 T (600.16 MHz). Standard processing parameters were used, and all spectra were processed in Topspin 3.5pl7.

#### Prediction of Sugar Composition Using Partial Least-Squares Models

Bruker’s AMIX software was used to divide spectra into 0.005 ppm buckets in the region of 3.10–4.15 ppm. The methanol peak, a byproduct of hydrolysis and centered at 3.37 ppm, was subtracted from all spectra both for models and predictions. PLS models were constructed from a model sample set using HPLC calculated sugar concentrations from hydrolysates of thirty-four samples from 12 biomass feedstocks (alfalfa, bagasse, corn stover, eucalyptus, fescue, guayule, miscanthus, pine, poplar, sunflower, switchgrass, and wheat straw) and performed in the Unscrambler v. 10.5 (CAMO A/S, Trondheim, Norway).

#### Pyrolysis Molecular Beam Mass Spectrometry (Py-MBMS) Analysis

Py-MBMS analysis was conducted as described previously.^27,41,42^ A Frontier PY2020 unit pyrolyzed 4 mg of destarched and ethanol extracted biomass samples at 500 °C for 30 s in 80 μL deactivated stainless steel cups. An Extrel Super-Sonic MBMS Model Max 1000 was used to collect mass spectral data from *m*/*z* 30 to 450 at 17 eV which was processed using Merlin Automation software (V3) and The Unscrambler X (V10.5). Lignin content and monomeric ratios were estimated as described previously (refs above) based on relative responses from standards of known Klason lignin content.^22,27,41,42^

#### Switchgrass Yield and Cost

We used individual plant biomass yield data collected at the three common garden field trial sites to estimate the commercial-scale per-area yield potential associated with the 331 different switchgrass genotypes in the natural variant population. For each genotype, we derived a single representative yield estimate for the broad southeastern U.S. region covered by the common garden sites. In gardens with multiple replicates subjected to different treatments, only the control treatment data (2 replicates in Knoxville, three in Tifton, and one in Watkinsville) were used. Since switchgrass and similar perennial grasses require approximately three seasons of growth in order to fully establish and reach their maximum yield potential,^30^ we focus exclusively on the year 3 biomass data for the rest of the analysis. At the time of analysis, three years of growth data were available for three replicates at the Tifton common garden site, two replicates at the Knoxville site, and a single replicate at the Watkinsville site. This assumes switchgrass yield is constant after 3 years before declining at the end of the life of a stand.^43^ The use of only year 3 yield data and the variation between sites lower the ability to make an absolute ranking of genotypes from these results. We excluded from further analysis any genotype that experienced mortality in any replicate at any common garden site or showed a plant biomass yield coefficient of variation greater than one between replicates at any individual site to focus on genotypes that are best adapted to the region.

#### Planting Density Correction

In breeding plots, individual switchgrass plants are typically planted in a grid or hexagon pattern at densities of less than 5 plants per square meter (m^–2^) to facilitate phenotyping. However, for commercial production, switchgrass is usually broadcast-seeded in dense swards, with tens or hundreds of plants m^–2^. Planting density affects total per-area yields, and the relative performance of different switchgrass varieties will change depending on whether they are planted at low or high density.^7^ The Knoxville, Watkinsville, and Tifton common garden sites are planted at densities of 0.17, 0.22, and 1.20 plants m^–2^, respectively. We multiplied the year 3 per-plant biomass data from each site by the site planting density to translate the observed yields to a per-area basis. Next, we calculated a weighted average per-area yield for each genotype across all three sites, weighting the Tifton data three times higher than that from Knoxville and Watkinsville, because the Tifton planting density was much closer to a commercially relevant density. We also applied a factor of 1.4 to represent the higher switchgrass yields in densely planted swards compared to spaced breeding plots (planting density of ∼1.3 plants m^–2^), based on data from four upland and four lowland varieties reported in Casler et al.^7^ The genotypes with the highest projected commercial-scale yield potential (35 and 39 dry Mg/ha) also achieved the highest yields at the more densely planted Tifton site (which was weighted more heavily in scaling).

#### Commercial-Scale Losses Correction

Per-area yield rates for commercial-scale energy crop production on marginal land are likely to be lower than that inferred from breeding plots due to plot edge effects, land quality, imperfect management, and biomass losses during mechanized harvest and bailing. Searle and Malins (2014)^4^ estimates yield rates of 2–10 Mg/ha for commercial-scale switchgrass production on marginal lands in temperate and warm temperature climate zones where most US production might occur. This range is approximately one-half of the yield range simulated by Lee et al.^44^ for the DOE Sun Grant Initiative Regional Feedstock Partnership using a hybrid model and expert judgment approach and the yield range compiled by McLaughlin et al.^45^ for a single-cut switchgrass systems across the sites of the DOE Bioenergy Feedstock Development Program. Thus, we apply a final correction factor of 0.5 to translate from plot-scale yield rates to the future commercial-scale yield potential. This value is also consistent with the guidance of Mola-Yudego et al.^46^ for estimating near-term commercial poplar yield expectations based on small plot data.

#### Harvest and Logistics Cost Model

To estimate the cost of switchgrass delivered to the biorefinery, a simulation model of the switchgrass supply chain (harvest, transport, storage, and grinding) was constructed in ExtendSim (Imagine That Inc., San Jose, CA, USA) by using the IBSAL 2.0 framework. The refinery feedstock requirement was assumed to be 2000 dry U.S. tons per day with a 3% dry matter loss (DML) during storage.^47^ The harvest window was assumed to be 120 days. Our switchgrass harvest process included mowing, baling with a large square baler, and in-field transport to the field side. The estimated processing rate and estimated cost per hour for each operation can be found in Table S8. All hourly costs included fuel and labor. Additional parameters for the feedstock supply and logistics cost models are given in Tables S8 and S9. These include estimates on the range the switchgrass is drawn from and the density of switchgrass fields around the biorefinery.

The delivered feedstock cost included not only the harvest and transport cost from the switchgrass discrete event simulation model but also land rental, planting, land maintenance, storage, and grinding as shown in Table S9. We assumed the crop would be planted on marginal nonirrigated pastureland. We assumed a hybrid storage system near the refinery where 50% of the feedstock would be tarped on a gravel pad and the remaining 50% would be stored in a pole barn.^48^ We also assumed the refinery would have limited feedstock storage of less than a week’s worth of inventory while it was being processed through a grinder. Total reactor-throat feedstock costs could then be expressed as a function of per-area switchgrass yield rates. This cost model was then interpolated to estimate the production cost associated with the commercial-scale yield rates estimated previously for each individual genotype. We excluded genotypes with a projected commercial-scale yield potential of less than 7.5 Mg/ha from further analysis, as these varieties would likely be uneconomical to produce (i.e., biomass costs greater than $115 Mg^–1^).

#### Techno-Economic Analysis

A subset of switchgrass samples from the natural variant common garden with complete compositional data for carbohydrates, lignin, and ash, feedstock yield model data, and feedstock cost model data was used in techno-economic analysis (n = 84). The compositional mass fractions of glucose, xylose, galactose, arabinose, and lignin from ^1^H NMR and lignin from py-MBMS were normalized by a factor of 0.95 to account for the destarched and ethanol extracted biomass used in compositional analysis. Where compositional data was not available, mannose, acetate, sucrose, and protein mass percentage were assumed to be 0, 2.00, 0, and 3.10 wt %, respectively. The ash content ranged from 1 to 5 wt % but was not considered further in this analysis. To achieve 100% mass closure, nonethanol soluble extractives were varied to complete the mass balance.

The process conversion TEA/LCA is based on a straightforward conversion model based on corn stover.^49^ We note that the MSFP or LCA values presented should be used for comparison of genotypes and of parameters or assumptions for further study–not for an absolute projected cost. Some other studies have performed similar analysis with actual lab-conversion data for single feedstocks.^13,50^ A limitation of this study is the assumption that the prior corn stover conversion models can be applied to switchgrass. Humbird et al.^49^ estimated an MSFP of ∼$0.57/L from stover which compares reasonably to our estimates. Much focus can fall upon the absolutes of the MFSP, compared to current energy costs, alternative fuel production pathways, or the ultimate potential of a process. In the context of this study, however, in efforts to understand the qualitative impacts of feedstock supply chain logistics versus compositional variation, the relative variation of the MFSP provides valuable insights into the biggest cost drivers in the process and where research focus may be placed to optimize economics.

For each sample, modified compositional data as described above were inputted into an Aspen-Plus model for cellulosic ethanol production for thermodynamically rigorous mass and energy balance calculations surrounding the process. This Aspen-model is updated from Humbird^49,51^ and consisted of dilute acid pretreatments followed by simultaneous saccharification and fermentation by yeast with added cellulolytic enzymes. Conversion rates and yield for each carbohydrate were based on corn stover data and varied from about 0.7 for arabinose to 0.9 for glucose. Galactose was not fermented in this model. In this model, lignin residuals are used for heating. Since the potential impact of lignin quality (e.g., S/G ratio) is not included in this conversion model, we do not make assumptions in current models that the S/G ratio impacts yields of sugars. Therefore, other compositional phenotypes (i.e., lignin quality) only indirectly influence the overall yield by their effect on the total fermentable carbohydrates. Resultant material and energy flows from the simulation and feedstock cost data were used in economic calculations to solve a discounted cashflow rate of return analysis for an ethanol minimum fuel selling price using methods and economic assumptions similar to those described in our previous work.^22^ Additionally, total material and energy inputs and outputs to and from the biorefinery were used to generate a life-cycle inventory for life cycle assessment (methods described below). Process ethanol yields were normalized to the per Mg basis using the volumetric ethanol output of the biorefinery divided by the total switchgrass feed. Field ethanol yields were calculated by multiplying the process ethanol yield by the feedstock yield per hectare.

### Life Cycle Assessment

#### Goal and Scope

The goal of this study is to assess the variability in ethanol GWP, CED, and AWARE due to inherent variability in switchgrass on-field yield and fermentable carbohydrate mass fraction based on a natural variant switchgrass population. We used these three LCA model outputs (GWP, CED, and AWARE) as estimates of “sustainability” of different genotypes in a biorefinery context. Obviously, many more aspects of sustainability can be considered. While these GWP estimates are lower than the range of those for gasoline, the goal is not to compare impacts associated with ethanol produced from switchgrass to other biofuels or to fossil fuels for the purpose of choosing a less impactful feedstock or biorefinery.

**Figure 10:**
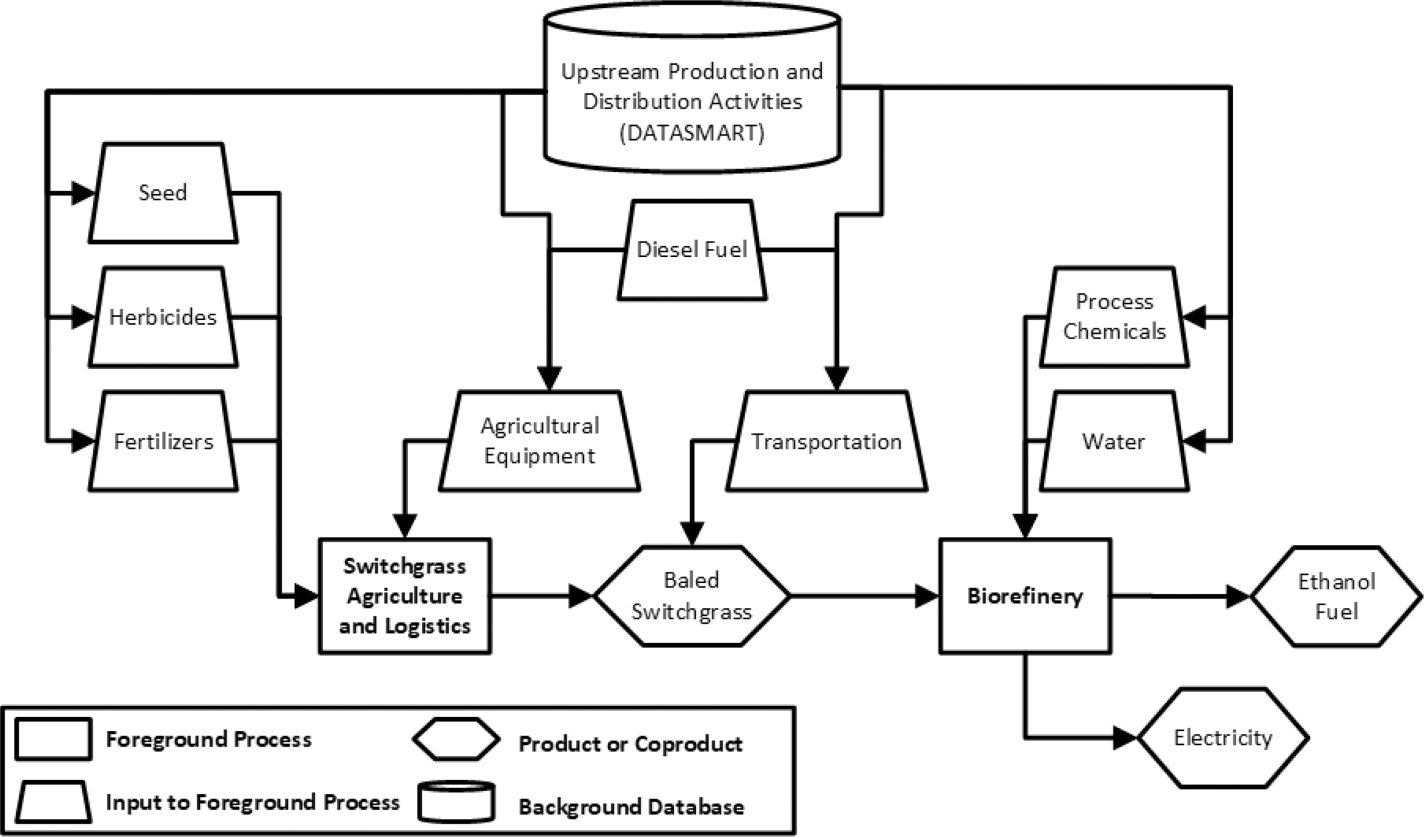
System boundary diagram for the switchgrass-to-ethanol life cycle. Transportation is included throughout the life cycle, although it is not shown explicitly, except for switchgrass transportation.

The scope of this study is the cradle-to-biorefinery-gate, and a system boundary diagram is given in Figure 10. All agricultural and logistical operations for a 10-year switchgrass rotation are within scope, including site preparation, cultivation, and harvesting and baling. Operations following the final harvest of the rotation that could prepare the land for a second rotation or for another land use type are not included in the scope. Long-term switchgrass storage operations are excluded from the scope. At the biorefinery, all material and energy inputs and coproducts are within scope. Excess electricity sold to the grid by the biorefinery is included as a coproduct, and energy-based allocation is applied to attribute impacts to the ethanol product. The ethanol use phase is excluded from the scope; thus, emissions released during ethanol combustion are not included. Biogenic emissions from the biorefinery and any carbon or greenhouse gas uptake or releases by switchgrass agriculture are not included in the scope. Impacts from direct and indirect land use change are not included in the scope.

#### Inventory Development

The functional unit of this study is 1 L of ethanol fuel produced from switchgrass.

The foreground life cycle inventory was developed from three main data sources. Agricultural operations and material and energy inputs follow Field et al.,^52^ with fuel consumption for yield-dependent operations and biomass logistics from the harvest and logistics cost model also used in the TEA. Material and energy inputs to the biorefinery for each switchgrass composition were obtained from Aspen simulations performed for the TEAs in this study and are summarized in Table S5. The background database used was the DATASMART life cycle inventory package, which combines Ecoinvent v2 process data with U.S.-based electricity grid information.^53^ Additional information about the foreground inventories for switchgrass agriculture is given in Table S4.

#### Simplifications, Proxy Data, and Assumptions

Grass seed production is used as a proxy for switchgrass seed production, for which data were not available. The grass seed transportation distance was assumed to be 161 km (100 miles) and to take place in the southeast United States. Switchgrass yield was originally provided as annual data (dry Mg/ha-year). Yield values were assumed to apply to all 9 years of the rotation in which harvesting takes place. Finally, application of the two herbicides is assumed to take place simultaneously; that is, a single pass of the field sprayer is assumed to apply both herbicides.

#### Impact Analysis

The impacts calculated for this study were 100-year global warming potential (GWP) as g CO_2_e/L ethanol, cumulative energy demand (CED) as MJ/L ethanol, and the Available Water Remaining (AWARE) indicator as m^3^ water/L ethanol.^23^ GWP was calculated by excluding all biogenic emissions from the biorefinery and carbon sequestration and uptake by switchgrass agriculture.

## Supporting Information Available

Nine tables and seven figures as called out in the text: Table S2 contains the data used in Figures 6, 7, 8, 9 and Figure S6. Figure S7 plots MSP versus carbohydrate for only the highest yield genotypes. Additional details of the feedstock cost model are in Figure S1, Table S8, Table S9; input parameters for the TEA and LCA are in Table S4, Table S5. Process contributions and statistics are in the other tables and figures. In addition, a data doi of intermediate data used in the figures and tables has been created at 10.25983/2217356 (PDF)
