# Supplementary material for: Economic and Sustainability Impacts of Yield and Composition Variation in Bioenergy Crops: Switchgrass (Panicum virgatum L.)
Source: ACS Sustain Chem Eng. 2024 Jan 22;12(5):1897–910. doi: 10.1021/acssuschemeng.3c05770 (PMC10848292; doi:10.1021/acssuschemeng.3c05770)
Supplement: Supplementary file 1 — sc3c05770_si_001.pdf [file sc3c05770_si_001.pdf]

Supporting Information

Economic and Sustainability impacts of yield and composition variation in bioenergy crops: *Panicum virgatum L.*

Renee M. Happs[1,10], Rebecca J. Hanes[2,10], Andrew W. Bartling[3,10], John L. Field[4,10], Anne E. Harman-Ware[1,10], Robin J. Clark[4,10], Thomas H. Pendergast IV[5,6,7,10], Katrien M. Devos[5,6,7,10], Erin G. Webb[4,10], Ali Missaoui[5,6,10], Yaping Xu[8,10], Shiva Makaju[5,6,10], Vivek Shrestha[8,10]†, Mitra Mazarei[8,10], Charles Neal Stewart, Jr.[8,10], Reginald J. Millwood[8,10] and Brian H. Davison[9,10]*

[1]Renewable Resources and Enabling Sciences Center, National Renewable Energy Laboratory; [2]Strategic Energy Analysis Center, National Renewable Energy Laboratory, Golden CI USA; [3]Catalytic Carbon and Transformation Center, National Renewable Energy Laboratory; [4]Environmental Sciences Division, Oak Ridge National Laboratory, Oak Ridge TN USA; [5]Institute of Plant Breeding, Genetics and Genomics, University of Georgia, Athens, GA 30602, USA; [6]Dept. Of Crop and Soil Sciences, University of Georgia, Athens, GA 30602, USA, [7]Dept. of Plant Biology, University of Georgia, Athens, GA 30602, USA [8]Dept. of Plant Sciences, University of Tennessee Knoxville, Knoxville TN USA, [9]Biosciences Division, Oak Ridge National Laboratory, Oak Ridge TN USA, [10]Center for Bioenergy Innovation, Oak Ridge National Laboratory, Oak Ridge TN USA.

†current affiliation: Bayer Crop Sciences, Bayer R&D, Chesterfield MO USA

*Corresponding Author: davisonbh@ornl.gov

Supporting information contains nine tables and seven figures over fourteen pages with 12 additional references.

Switchgrass Yield and Cost

Figure S1 shows the results of the feedstock cost model as a function of commercial-scale switchgrass yield, for a fixed annual biorefinery demand and density of fields in switchgrass production. The components of total delivered switchgrass costs include marginal land rental, planting, annual land maintenance, harvest, transport, storage, and grinding. Harvest, transport, and land maintenance costs decrease the most as the switchgrass yield increases from 5 to 17 Mg ha^{-1}. An increase in yield reduces the land requirement which affects the harvest and land maintenance cost while the reduced supply shed footprint affects the transport cost. However, total costs become much less sensitive to yield above 18 Mg ha^{-1}, with the rate of change in total cost per megagram when increasing the yield by 1 megagram per hectare is less than two percent. It should be noted that this is not the price that a biorefinery would pay for feedstock at the refinery gate, but rather is a techno-economic cost that would be incurred if the refinery managed the feedstock supply chain up to the beginning of the conversion processes or the first "reactor throat" or the beginning of the conversion processing. We can assume that if the refinery was purchasing the feedstock from local growers, then the purchase cost would be based on the incurred cost plus a factor for grower profit.

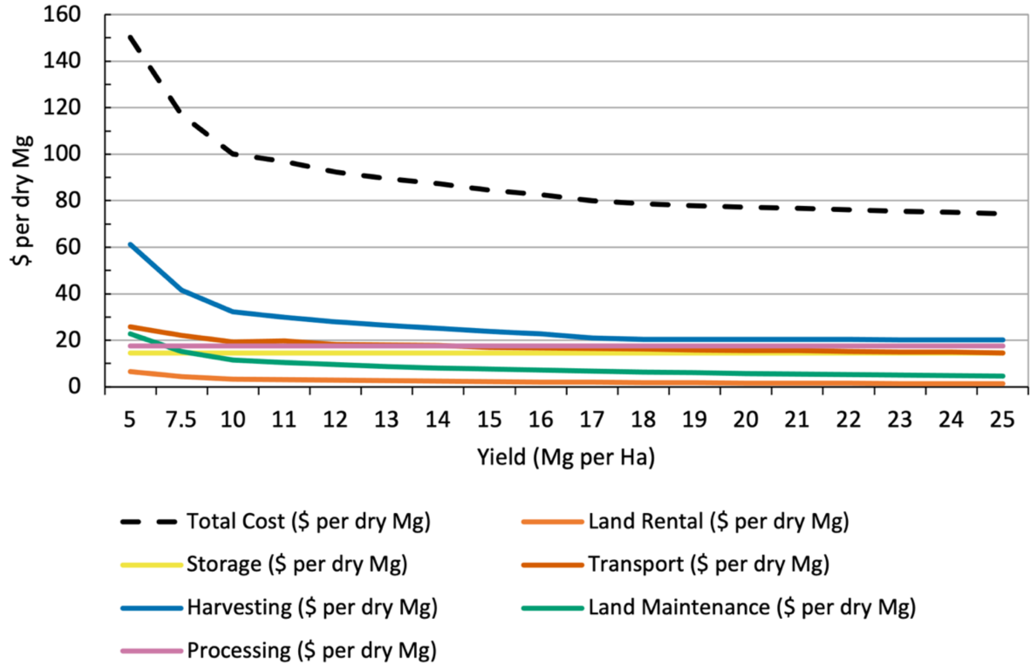

Figure S1. Feedstock delivered costs ($ per dry Mg) as a function of switchgrass yield.

Composition

Table S1. Percent Composition Statistics for 331 analyzed switchgrass samples selected from the population (% w/w)

	Glucose	Xylose	Galactose	Arabinose	Lignin	S/G Ratio
**Average**	40	25	1.4	2.5	18.8	0.68
**Minimum**	35	22	0.8	1.4	15.4	0.47
**Maximum**	46	28	2.5	4.1	21.4	0.92

**Prediction Error**	±3	±2	±0.6	±0.4	N/A*	N/A*
**Std. Dev.**	±2	±1	±0.3	±0.4	±0.96	±0.02

** Lignin and syringyl/guaiacyl ratio (S/G) were not determined via models but predicted relative to standard response, hence no model error reported, see Decker et al., 2018* for analysis details[1].

Key Numerical Results for Figures 6, 7, 8, 9 and S6.

Table S2. Life cycle GWP, CED, and AWARE results for all variants, alongside the fermentable carbohydrate mass fraction, on-farm yield, and MFSP values.

VARIANT	GWP (g CO_2e/L)	CED (MJ/L)	AWARE (m^3/L)	FERM. CARB. MASS FRACTION	YIELD (DRY MG/HA)	MFSP (USD/L)
**J324-A**	474	8.88	46.7	0.655	34.9	0.588
**J303-A**	549	10.0	49.1	0.659	9.28	0.674
**J594-C**	492	9.11	47.6	0.653	18.8	0.605
**J341-A**	540	9.82	48.7	0.651	9.95	0.669
**J271-A**	498	9.25	47.6	0.650	16.6	0.619
**J484-A**	492	9.18	47.3	0.658	18.3	0.607
**J016-B**	523	9.61	47.3	0.676	10.6	0.643
**RAMBO 4**	476	8.87	46.2	0.669	21.4	0.587
**J463-A**	466	8.70	46.2	0.658	39.2	0.577
**J496-C**	497	9.18	48.1	0.649	18.4	0.611
**J499-B**	508	9.35	48.1	0.655	14.7	0.624
**X211-A**	484	9.00	46.6	0.669	18.2	0.597
**X222-A**	477	8.92	46.7	0.661	26.5	0.590
**J319-A**	483	9.00	46.8	0.662	20.6	0.597
**J321-A**	493	9.11	47.3	0.667	16.7	0.604
**J497-B**	536	9.82	49.0	0.656	11.3	0.657
**J041-A**	542	9.84	48.7	0.655	9.62	0.670
**X502-A**	522	9.59	47.7	0.663	11.3	0.644
**J013-C**	533	9.71	48.6	0.651	11.0	0.655
**J212-A**	586	10.4	51.4	0.619	8.11	0.727
**J330-A**	474	8.87	46.1	0.673	24.0	0.583
**J022-B**	557	10.0	49.3	0.642	8.67	0.693
**X229-A**	537	9.74	48.8	0.643	10.3	0.667
**J280-A**	521	9.54	49.0	0.640	13.5	0.641
**J073-B**	500	9.28	47.3	0.657	14.9	0.620
**J295-A**	477	8.97	46.3	0.669	23.5	0.588
**J226-A**	516	9.48	48.6	0.643	14.0	0.635
**J251-B**	549	9.94	50.2	0.615	11.0	0.686
**J250-A**	585	10.5	50.4	0.641	7.63	0.722

**J419-A**	514	9.42	48.5	0.640	14.0	0.635
**J340-A**	510	9.40	49.0	0.633	16.8	0.631
**J218-A**	488	9.12	47.1	0.656	19.8	0.605
**J023-B**	554	10.0	50.3	0.619	10.5	0.690
**J514-A**	500	9.21	48.1	0.643	17.3	0.618
**J331-A**	567	10.2	49.4	0.648	7.89	0.701
**RAMBO 5**	501	9.19	48.9	0.621	21.0	0.621
**J274-A**	506	9.32	48.5	0.628	16.5	0.631
**J177-A**	507	9.26	49.4	0.615	19.2	0.631
**J315-A**	514	9.47	48.8	0.628	14.9	0.641
**J610-B**	505	9.28	48.1	0.647	15.1	0.624
**J249-B**	541	9.87	48.7	0.647	9.93	0.674
**J394-C**	531	9.67	48.4	0.645	11.0	0.658
**J008-B**	553	10.0	49.7	0.638	9.61	0.685
**J301-A**	487	9.03	47.2	0.656	20.4	0.599
**J065-A**	490	9.10	47.2	0.656	18.5	0.605
**J251-C**	481	8.95	47.1	0.647	24.7	0.595
**J499-A**	517	9.54	49.4	0.632	15.8	0.638
**J272-A**	497	9.21	47.6	0.657	16.9	0.612
**J251-A**	470	8.85	45.3	0.688	20.8	0.578
**J610-C**	575	10.3	49.7	0.648	7.74	0.708
**J497-C**	497	9.25	48.0	0.654	19.3	0.611
**J004-B**	486	9.06	46.6	0.667	17.2	0.601
**J447-A**	580	10.4	50.3	0.638	7.76	0.717
**J249-A**	538	9.72	49.6	0.620	11.4	0.672
**J270-A**	554	10.0	48.3	0.669	8.13	0.684
**J247-A**	499	9.25	48.3	0.634	20.2	0.618
**J065-B**	510	9.41	48.1	0.645	14.4	0.633
**J016-D**	534	9.73	48.9	0.648	11.3	0.655
**J276-A**	548	10.0	49.0	0.648	9.49	0.680
**J022-A**	468	8.84	45.5	0.674	26.1	0.580
**J235-A**	559	10.2	48.1	0.676	7.64	0.688
**J250-C**	534	9.80	47.9	0.667	9.87	0.660
**J610-A**	546	9.93	48.7	0.662	9.22	0.674
**J016-A**	490	9.14	47.1	0.668	18.4	0.602
**J497-A**	497	9.21	47.4	0.663	15.9	0.612
**J005-D**	524	9.58	47.7	0.669	10.8	0.644
**J003-D**	515	9.49	47.5	0.666	11.9	0.636
**J326-A**	495	9.17	47.2	0.661	16.1	0.611
**J594-A**	558	10.1	48.8	0.660	8.25	0.690
**J215-A**	498	9.20	48.4	0.640	20.2	0.612
**J249-C**	483	8.97	47.1	0.647	24.2	0.597
**J237-A**	502	9.25	48.2	0.643	16.2	0.621

**J503-A**	577	10.3	51.7	0.616	8.92	0.714
**J294-A**	524	9.58	49.1	0.632	13.2	0.649
**J327-A**	559	10.1	49.7	0.644	9.05	0.690
**J020-C**	526	9.66	49.3	0.632	13.6	0.651
**J008-D**	534	9.78	49.5	0.633	12.2	0.661
**J268-A**	542	9.90	49.9	0.618	11.7	0.676
**J305-A**	514	9.43	48.8	0.630	14.7	0.639
**J322-A**	509	9.40	48.8	0.630	16.8	0.631
**J293-A**	562	10.2	50.7	0.611	9.90	0.705
**J001-A**	515	9.45	48.1	0.650	12.9	0.636
**J587-B**	484	9.02	47.1	0.653	22.7	0.599
**J323-A**	490	9.06	47.7	0.640	22.0	0.606

Statistical Summary of Numerical Results

Table S3. Statistical summary of ethanol life cycle impacts observed in this study.

	**GWP (Gco_2e/L)**	**CED (MJ/L)**	**AWARE (m^3/L)**
Mean	517	9.50	48.3
Minimum	466	8.70	45.3
Maximum	586	10.5	51.7
Standard Deviation	30.6	0.445	1.27

Inventory Comparison to Other Studies

Table S4. Agricultural inputs comparison between GREET (2021)[2], and this study, adapted from Supplementary Table 8 of Field et al (2018)[3]. Where values are given as ranges, the exact values used for a variant were dependent on the estimated switchgrass yield projected from the single plant yields. While the units here are reported in metric or English units as in the cited studies, all were converted in SI for the calculations.

**Agricultural Input**	**Units**	**GREET (2021)[2]**	**Field et al (2018)[3]**	**This Study**
Seed	kg/dry U.S. ton	-	-	0.030 – 0.110
N fertilizer	kg N/dry U.S. ton	4.30	5.67	5.51
P_2O_5 fertilizer	kg P/dry U.S. ton	0.221	3.56	1.88 – 2.28
K_2O fertilizer	kg K_2O/dry U.S. ton	2.80	15.0	11.9 – 12.5
$CaCO_3$ fertilizer	kg $CaCO_3$/dry U.S. ton	5.10	-	-
Atrazine	kg chemical/dry U.S. ton	0.0467 (generic herbicide)	0.066	0.021 – 0.069
2,4D	kg chemical/dry U.S. ton		0.267	0.113 – 0.368
Insecticide	kg chemical/dry U.S. ton	0	-	-

Farm operations diesel use	BTU/dry U.S. ton	5.94×10^4 (farming and harvest)	3.90×10^4	6.30×10^3 – 2.05×10^4
Harvest operations diesel use	BTU/dry U.S. ton		1.28×10^5	1.61×10^5 – 3.07×10^5

Table S5. Biorefinery inputs and coproduct comparison between GREET (2021)[2] and this study. Where values are given as ranges, the exact values used for a variant were dependent on switchgrass composition.

**Biorefinery Input**	**Units**	**GREET (2021)[2]**	**This Study**
Switchgrass, bone dry	kg/gal ethanol	11.3	9.56 – 10.8
Sulfuric acid		0.300	0.227 – 0.256
Ammonia		0.048	0.140 – 0.158
Corn steep liquor		0.136	0.155 – 0.175
Diammonium phosphate		0.015	0.017 – 0.019
Sodium hydroxide, 100%		0.103	0.271 – 0.304
Lime		0.065	0.085 – 0.111
Glucose		2.73×10^{-4}	0.305 – 0.343
Sulfur Dioxide		1.63×10^{-3}	2.07×10^{-3} – 2.33×10^{-3}
Sorbitol		4.50×10^{-3}	5.14×10^{-3} – 5.78×10^{-3}
Host Nutrients		6.88×10^{-3}	0.008 – 0.010
Boiler Chemicals		-	2.81×10^{-5} – 3.27×10^{-5}
Cooling Tower Chemicals		2.50×10^{-4}	2.41×10^{-4} – 2.97×10^{-4}
Makeup water		-	15.8 – 19.5
Electricity coproduct	kWh/gal ethanol	1.79	0.878 – 1.95

Process Contribution Analysis

A process contribution analysis (PCA) provides more detailed insight into environmental hotspots, which are processes involved in ethanol production which may be producing a disproportionate level of impacts. This analysis can be a first step towards identifying and prioritizing research areas that could reduce total life cycle impacts. For this study, the objective of the PCA is to identify any trends that may exist in process-specific impacts due to variability in FCMF or in switchgrass yield. Variants were selected for PCA based on five percentiles of the FCMF values and of the yield values represented in the study. After calculating the FMCF percentiles, the five variants with FCMF values closes to those percentiles were selected for PCA, and five variants representing the switchgrass yield percentiles were chosen analogously. Figure S2 shows box-and-whisker plots of the five percentiles for FCMF (A) and for yield (B), with percentiles labeled with the corresponding variant names in gray.

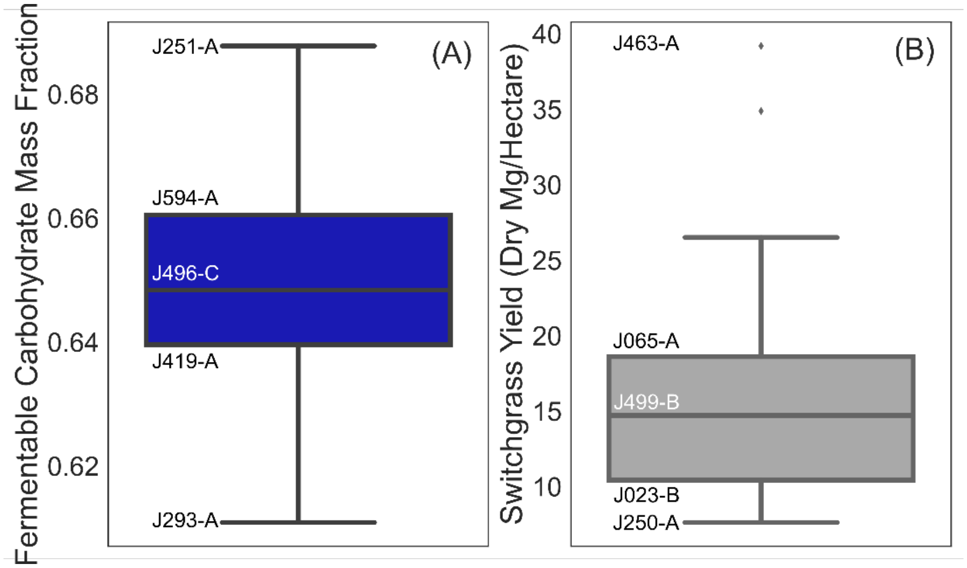

Figure S2. Box-and-whisker plots showing (A) five fermentable carbohydrate mass fraction percentiles and (B) five switchgrass yield percentiles, with the switchgrass variants nearest to each percentile labeled in gray. The process contribution analysis was performed on these ten variants.

The ethanol life cycle was divided into five process categories for the PCA. Four of these categories cover switchgrass agriculture and logistics: site preparation and planting, chemicals and application operations, harvest operations, and transport to biorefinery. The final category consists of inputs to the biorefinery.

PCA results are shown in Figures S3, S4, and S5 for GWP, CED, and the AWARE indicator, respectively. Numerical PCA results are listed in Tables S6 and S7. In each PCA figure, results for the FCMF percentile variants are given in sub-figure (A) and the yield percentile variants are in sub-figure (B). A lighter shade indicates a higher percentile. For all three impacts, increased FCMF does not correspond to a uniformly increased or decreased impact in any of the five process categories, while increased yield does correspond to decreased impacts for all categories except site preparation and planting.

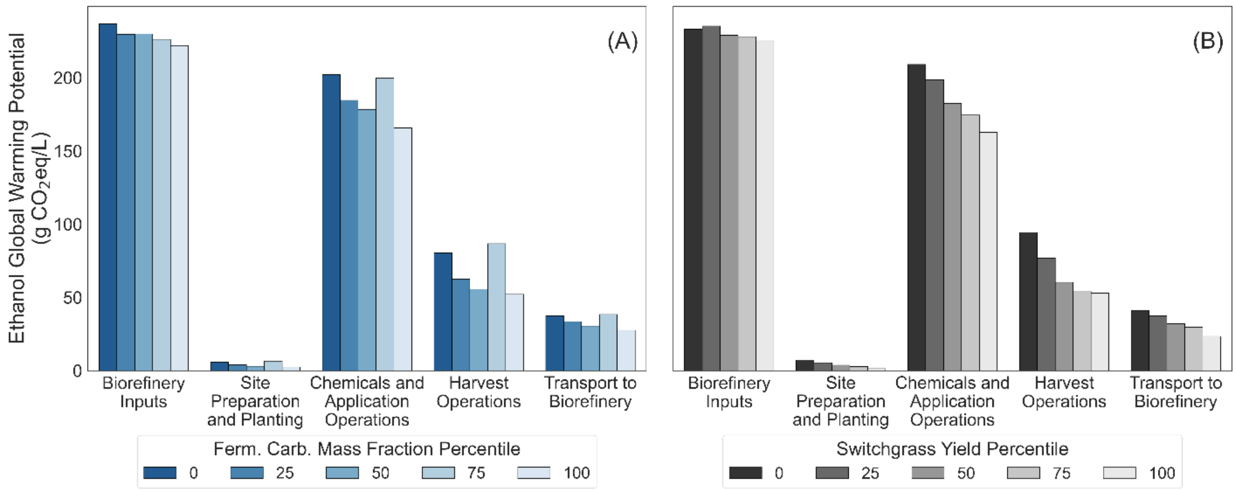

Figure S3. Global warming potential for 1 L ethanol disaggregated by process category and shown for selected variants based on carbohydrate fraction percentiles (A) and yield percentiles (B).

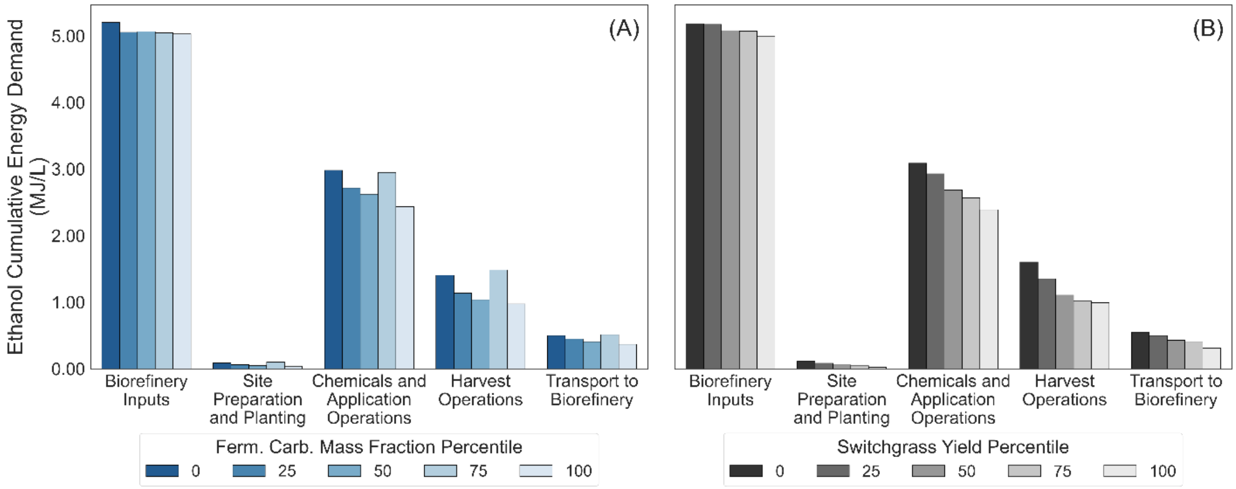

Figure S4. Cumulative energy demand for 1 L ethanol disaggregated by process category and shown for selected variants based on carbohydrate fraction (A) and yield percentiles (B).

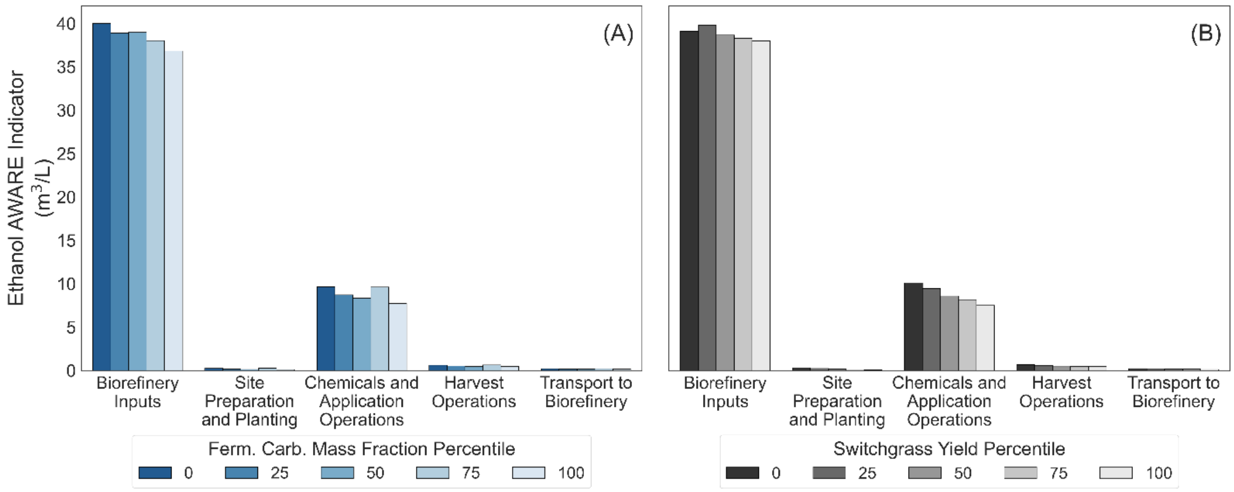

Figure S5. Available water remaining indicator for 1 L ethanol disaggregated by process category and shown for selected variants based on carbohydrate fraction (A) and yield percentiles (B).

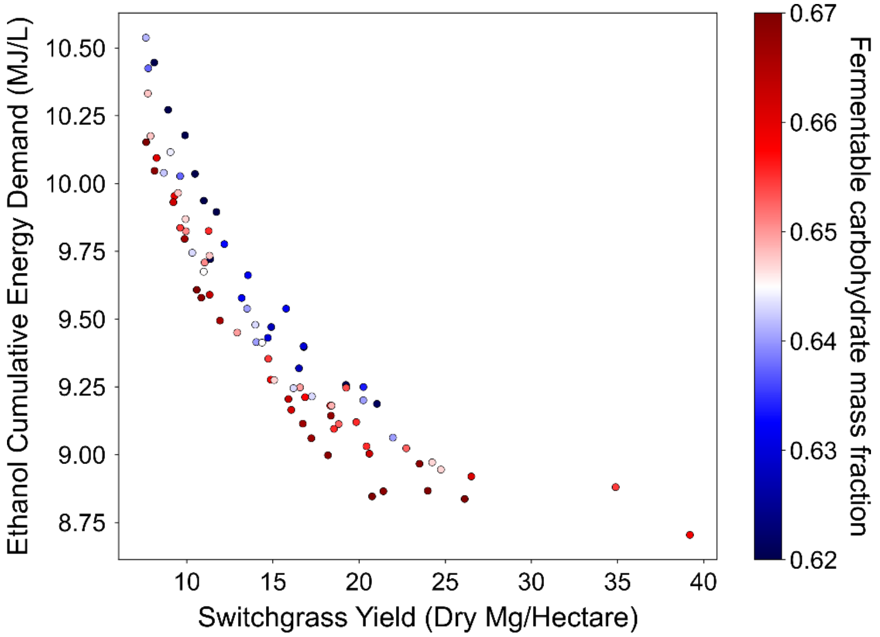

Figure S6. Ethanol cumulative energy demand (MJ/L) shows a strong negative correlation (R^2 = 0.77) with switchgrass yield.

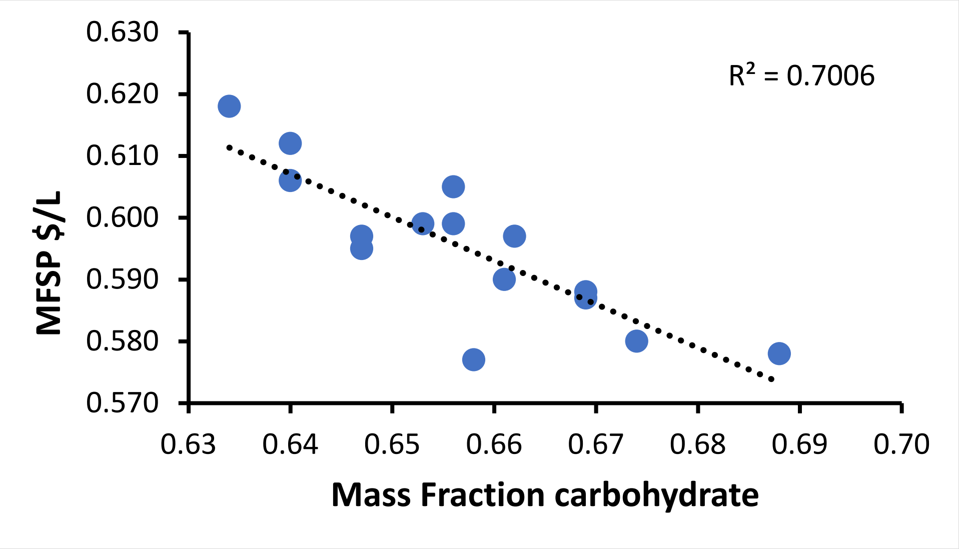

Figure S7: MFSP decreases with increasing fermentable carbohydrate content. (R^2 = 0.70) when considering only the higher yielding switchgrass genotypes (≥ 20 dry Mg/ha)

Table S6. Process contribution analysis results for the fermentable carbohydrate mass fraction percentile variants.

	J293-A (0th)	J419-A (25th)	J496-C (50th)	J594-A (75th)	J251-A (100th)
**Global Warming Potential (g CO_2e/L)**					
Biorefinery Inputs and Co-Products	237	230	230	226	222
Site Preparation and Planting	5.75	3.94	3.01	6.55	2.51
Chemicals and Application Operations	202	185	178	200	166
Harvest Operations	80.4	62.4	55.6	86.7	52.1
Transport to Biorefinery	37.4	33.5	30.5	38.4	27.6
**Cumulative Energy Demand (MJ/L)**					
Biorefinery Inputs and Co-Products	5.20	5.05	5.07	5.05	5.03
Site Preparation and Planting	0.088	0.0603	0.0461	0.100	0.0385
Chemicals and Application Operations	2.98	2.72	2.62	2.95	2.44
Harvest Operations	1.40	1.14	1.04	1.49	0.974
Transport to Biorefinery	0.500	0.448	0.408	0.513	0.369
**AWARE (m^3/L)**					
Biorefinery Inputs and Co-Products	40.0	38.9	39.0	38.0	36.8
Site Preparation and Planting	0.249	0.170	0.130	0.283	0.109
Chemicals and Application Operations	9.66	8.71	8.35	9.62	7.74
Harvest Operations	0.620	0.523	0.487	0.641	0.454
Transport to Biorefinery	0.206	0.185	0.168	0.212	0.152

Table S7. Process contribution analysis results for the switchgrass yield percentile variants.

	**J250-A (0th)**	**J023-B (25th)**	**J499-B (50th)**	**J065-A (75th)**	**J463-A (100th)**
**Global Warming Potential (g CO_2e/L)**					
Biorefinery Inputs and Co-Products	233	235	229	228	225
Site Preparation and Planting	7.28	5.40	3.73	2.93	1.38
Chemicals and Application Operations	209	199	182	175	163
Harvest Operations	94.4	76.9	60.4	54.4	52.8
Transport to Biorefinery	41.2	37.3	32.1	29.7	23.4
**Cumulative Energy Demand (MJ/L)**					
Biorefinery Inputs and Co-Products	5.18	5.17	5.08	5.07	4.99
Site Preparation and Planting	0.111	0.0827	0.0572	0.0448	0.0211
Chemicals and Application Operations	3.09	2.93	2.68	2.57	2.39
Harvest Operations	1.60	1.35	1.11	1.02	0.991
Transport to Biorefinery	0.551	0.499	0.429	0.398	0.313
**AWARE (m^3/L)**					
Biorefinery Inputs and Co-Products	39.1	39.8	38.7	38.3	38.0
Site Preparation and Planting	0.315	0.233	0.161	0.127	0.0596
Chemicals and Application Operations	10.1	9.47	8.59	8.17	7.51
Harvest Operations	0.685	0.601	0.512	0.477	0.452
Transport to Biorefinery	0.227	0.206	0.177	0.164	0.129

Feedstock Supply Model Details

Our switchgrass transport process included crews of semi-trucks with flatbed trailers moving the bales to storage near the refinery with loaders at both the field and the storage facility. The storage near the refinery consisted of a hybrid storage process with half of the feedstock stored under a tarp on a gravel pad and the other half stored covered under a pole barn. The tarped feedstock would be used first so weather during storage would have minimal effect on the stored feedstock. The system would only keep a few days' supply of feedstock at the refinery where it is processed by a grinder.

Table S8. Feedstock cost model parameters. While the units here are reported in metric or English units as in the cited studies, all were converted in SI for the calculations.

Description	Value
Dry matter loss (DML) during storage	3% [4]
Bale Data (dimension; density; moisture)	8x4x3 ft^3 [5]; 175 dry Kg per m^3 [5];15% [6]
On highway diesel	$4.10 per gallon [7]
Ag Equipment Operator Labor Rate	$15.63 per hour [8]
Semi Transport Labor Rate	$22.66 per hour [8]
Self-Propelled Mower Conditioner	1.8 ha per hour [9];31.2 mg per hour [9]; $175.3 per hour[10,a]
Tractor and Large Square Baler	1.4 Ha per hour [9]; 25.1 dry Mg per hour [9,11]; $226.5 per hour [10, b]
Move to field side	72 bales per hour [c]; 34.3 dry mg per hour [5]; $148.1 per hour [10, d]
Transport Crew with three to four Semi-trailer trucks with flatbed trailers and a loader at the field side	36 Bales per load; 17.1 dry mg per load [5, e]; $208.89 [f,g]

[a] Using a 235 hp mower conditioner (JD W235M) with a list price of $184,457

[b] Using a large square baler (JD L341) with a list price of $198,583 pulled by a 190 hp tractor (JD 6R 195) with a list price of $247,674.

[c] as suggested by FDC Enterprises.

[d] Assuming a purchase cost of $202,500 for a 320 hp Stinger Stacker 6500.

[e] Assuming a bale density of 175 dry Kg per m^3 and a moisture of 15%.

[f] Assuming a purchase cost of $109,835 for a semi-truck, $49,410 for a flatbed trailer (53 ft. X 102 in.), and $85,112 for a wheeled loader (JD 204L Wheeled Loader).

[g] Using one loader at the field for each transport crew consisting of three to four semi-trucks with flatbed trailers.

The number of fields required was calculated for each yield scenario using the annual refinery demand of 2000 dry U.S. tons while assuming a 3% dry matter loss (DML) during storage (3), a field size of 60 hectares, and the switchgrass yield set by the scenario which ranged from 5 to 40 Mg ha^{-1}. We assumed a switchgrass supply shed density of 15% which would randomly place one 60-hectare field every four km^2. The road distance from the refinery to the field was assumed to be a factor of 1.4 times the direct distance.

Table S9. Feedstock cost model parameters

Description	Value
Land Rental	$32.12 per hectare [12]
Planting and Annual Land Maintenance	$110.56 per hectare per year [13]
Storage Cost	$14.43 per dry megagram [14]
Short Term Storage at Refinery	$1.8 per dry megagram [13]
Grinding	$17.66 per dry megagram [13]

References for Supplemental Information only:

1. Decker, S.R.; Harman-Ware, A.E.; Happs, R.M.; Wolfrum, E.J.; Tuskan, G.A.; Kainer, D.; et al. High Throughput Screening Technologies in Biomass Characterization. Frontiers in Energy Research [Internet]. 2018, 6(120). Available from: https://www.frontiersin.org/article/10.3389/fenrg.2018.00120

2. Wang, M.; Elgowainy, A.; Lee, U.; Bafana, A.; Banerjee, S.; Benavides, P.; Bobba, P.; Burnham, A.; Cai, H. ; Gracida, U.; Hawkins, T.; Iyer, R.; Kelly, J.; Kim, T.; Kingsbury, K.; Kwon, H.; Li, Y.; Liu, X.; Lu, Z.; Ou, L.; Siddique, N.; Sun, P.; Vyawahare, P.; Winjobi, O.; Wu, M.; Xu, H.; Yoo, E.; Zaimes, G.; Zang, G. Greenhouse gases, Regulated Emissions, and Energy use in Technologies Model ® (2021 Excel) [Internet]. Argonne National Laboratory (ANL), Argonne, IL (United States); 2021 [cited 2023 Jun 20]. Available from: https://www.osti.gov/doecode/biblio/63044. DOI 10.11578/GREET-EXCEL-2021/DC.20210902.1.

3. Field, J.L.; Evans, S.G.; Marx, E.; Easter, M.; Adler, P.R.; Dinh, T.; Wilson, B.; Paustian, K. High-resolution techno–ecological modelling of a bioenergy landscape to identify climate mitigation opportunities in cellulosic ethanol production. Nat Energy. 2018, 3(3):211–9. DOI 10.1038/s41560-018-0088-1.

4. Darr, M.J.; Shah, A. Biomass storage: an update on industrial solutions for baled biomass feedstocks. Biofuels. 2012, 3(3):321–32. DOI 10.4155/bfs.12.23.

5. Shinners, K.; Friede, J. Energy Requirements for Biomass Harvest and Densification. Energies. 2018, 11(4):780. DOI 10.3390/en11040780.

6. Hess, J.R.; Wright, C.T.; Kenney, K.L.. Cellulosic biomass feedstocks and logistics for ethanol production. Biofuels, Bioprod Bioref. 2007, 1(3):181–90. DOI 10.1002/bbb.26.

7. EIA. Gasoline and Diesel Fuel Update [Internet]. U.S. Energy Information Administration; 2020 Feb. Available from: https://www.eia.gov/petroleum/gasdiesel/

8. BLS. Employer costs for employee compensation news release [Internet]. Washington, D.C.: U.S. Bureau of Labor Statistics; 2020. Available from: https://www.bls.gov

9. Mathanker, S.K.; Hansen, A.C. Impact of miscanthus yield on harvesting cost and fuel consumption. Biomass and Bioenergy. 2015, 81:162–6. DOI 10.1016/j.biombioe.2015.06.024.

10. Turhollow, A.F.; Wilkerson, E.G.; Sokhansanj, S. Cost methodology for biomass feedstocks herbaceous crops and agricultural residues. Oak Ridge National Laboratory, Oak Ridge, TN; 2009. Report No.: ORNL/TM-2008/1052009.

11. Shinners, K.J.; Boettcher, G.C.; Muck, R.E.; Weimer, P.J.; Casler, M.D. Harvest and Storage of Two Perennial Grasses as Biomass Feedstocks. Transactions of the ASABE. 2010; 53(2):359–70. DOI 10.13031/2013.29566.

12. USDA/NASS. Land values and cash rents, 2020 summary [Internet]. Washington, D.C.: US Department of Agriculture, National Agricultural Statistics Service; 2020. Available from: https://www.nass.usda.gov/Publications/Highlights/2020/land-values-cash-rents.pdf

13. U.S. Department of Energy. 2016 Billion-Ton Report: Advancing Domestic Resources for a Thriving Bioeconomy, Volume 1: Economic Availability of Feedstocks. Oak Ridge National Laboratory, Oak Ridge, TN; 2016. Report No.: ORNL/TM-2005/66.

14. Stauffer, H.; Richard, T.; Webb, E.; Field, J.; Clark, R. Optimizing Biomass Storage Facility Configuration to Minimize Supply Chain Risk and Vulnerability. 2023; Personal Communication 2023 June 06.
